# Oxygen Vacancy Defect Engineering for Transverse Thermoelectric Enhancement: a Novel Extrinsic Pathway beyond Intrinsic Approaches

**DOI:** 10.1002/advs.202502892

**Published:** 2025-04-17

**Authors:** Min Young Kim, Dongkyu Lee, June Ho Lee, Donghwa Lee, Gi‐Yeop Kim, Si‐Young Choi, Joseph P. Heremans, Hyungyu Jin

**Affiliations:** ^1^ Department of Mechanical Engineering Pohang University of Science and Technology (POSTECH) Pohang 37673 South Korea; ^2^ Department of Mechanical and Aerospace Engineering The Ohio State University Columbus OH 43210 USA; ^3^ Department of Materials Science and Engineering Pohang University of Science and Technology (POSTECH) Pohang 37673 South Korea; ^4^ Center for Van der Waals Quantum Solids Institute for Basic Science (IBS) Pohang 37673 South Korea; ^5^ Department of Materials Science and Engineering The Ohio State University Columbus OH 43210 USA; ^6^ Department of Physics The Ohio State University Columbus OH 43210 USA

**Keywords:** anomalous Nernst effect, defect chemistry, oxygen vacancy, thermoelectrics, variable‐range hopping

## Abstract

For efficient transverse thermoelectric (TE) generation, research has primarily focused on achieving high transverse TE conductivity by utilizing intrinsic material properties. While this approach remains fundamental, exploring extrinsic strategies, such as defect engineering, offers new opportunities to further enhance performance and expand the range of applicable materials. This study investigates the impact of oxygen vacancies—an extrinsic factor—on the anomalous Nernst effect, a key transverse TE mechanism, using disordered semiconducting Sr_3_YCo_4_O_11‐_ *_δ_* (*δ* = 0.02, 0.08 and 0.14) as a model system. The highest anomalous Nernst thermopower (*S*_ANE_) occurs at *δ* = 0.14, showing a 44% increase compared to *δ* = 0.02. This enhancement arises from two synergistic effects: i) increased Co^3+^/Co^4+^ mixed valency, boosting entropy‐driven charge transport and Seebeck thermopower, and ii) distortions in the Co‐O‐Co bond angle, elevating the local density of states and the anomalous Nernst angle. These findings establish oxygen vacancy defect engineering as a potent strategy for enhancing transverse TE performance, broadening the spectrum of viable TE materials for diverse engineering applications.

## Graphical abstract

Transverse thermoelectric (TE) technology gains increasing attention as it enables efficient power generation within a single TE element and eliminates junctions. This study enhances transverse thermopower in polycrystalline Sr_3_YCo_4_O_10‐_ *_δ_* through oxygen vacancy defect engineering, utilizing the anomalous Nernst effect to improve TE efficiency. These findings provide a new route for high‐performance transverse TE materials.

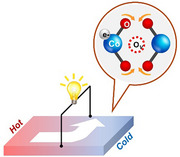

## 1 Introduction

**Figure 1:**
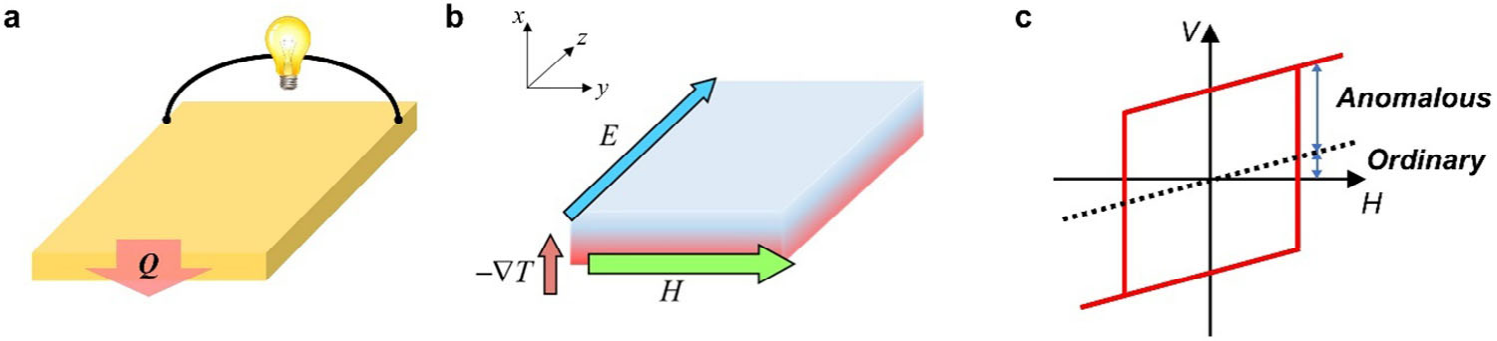
Description of transverse thermoelectric (TE) generation via the Nernst effect. a) A Nernst‐effect‐based transverse TE device consisting of a single TE element. (*Q*: heat flux) b) Schematic illustration of the measurement geometry for the Nernst effect. (∇*T*: temperature gradient, *H*: magnetic field and *E*: Nernst field) c) *H* dependence of Nernst voltage (*V*), which includes both ordinary and anomalous contributions.

Thermoelectricity is a promising technology for energy harvesting, enabling the conversion of heat into electricity through solid‐state devices. As thermoelectricity emerges as a key future energy solution, substantial efforts have been dedicated to developing efficient thermoelectric (TE) devices.^[^ ^1^, ^2^, ^3^, ^4^, ^5^, ^6^ ^]^ Notably, transverse TE conversion has attracted considerable attention due to its strong potential in practical applications.^[^ ^7^, ^8^, ^9^, ^10^, ^11^, ^12^, ^13^, ^14^, ^15^, ^16^ ^]^ For example, Nernst‐effect‐based TE devices, a prime example of transverse TE generators, can achieve efficiencies nearly four times higher than those of traditional Seebeck‐effect‐based devices, given the same figure‐of‐merit *ZT* of 1.^[^ ^17^, ^18^ ^]^ Furthermore, transverse geometry necessitates only a single element (**Figure** **1**a), which allows the output power (voltage) to be scaled by simply increasing the surface area (length) of the element perpendicular to the heat flux. It also avoids the need for electrical contacts on the hot side of the generator, enabling a junctionless structure and thereby enhancing device efficiency.^[^ ^12^ ^]^

The Nernst effect involves transverse TE generation facilitated by an external magnetic field or intrinsic remanent magnetization within a material.^[^ ^19^ ^]^ As described in Figure 1b, when a magnetic field (*H*) and a temperature gradient (∇*T*) are applied perpendicular to each other, thermally excited electrons acquire a velocity in the *z*‐direction and it generates a transverse Nernst field (*E*). Here, the ordinary Nernst voltage depends linearly on the magnitude of *H* under the influence of the Lorentz force while the anomalous Nernst voltage is unusually large even without the presence of a strong magnetic field (Figure 1c). Given this unique advantage, the anomalous Nernst effect (ANE) has become the primary focus of transverse TE research, driving the development of efficient ANE materials and devices and showcasing its potential to advance TE technology.^[^ ^20^, ^21^, ^22^, ^23^, ^24^, ^25^, ^26^, ^27^ ^]^

Particularly, topological materials stand out as promising candidates due to their intrinsically large transverse TE conductivity (*α_zx_*), governed by the Mott relation $\alpha_{zx}=\frac{\pi^{2}{k_{B}}^{2}}{3e}\;T\frac{\partial \sigma_{zx}}{\partial \varepsilon }{|}_{\varepsilon_{F}}$ (*σ_zx_*: transverse electrical conductivity and *ε*_F_: Fermi level).^[^ ^28^ ^]^ For instance, anomalous Nernst thermopowers of a few µV K^−1^ have been observed over different temperature ranges, including single‐crystalline Co_3_Sn_2_S_2_,^[^ ^29^, ^30^ ^]^ Co_3_MnGa,^[^ ^31^, ^32^ ^]^ Fe_3_Ga,^[^ ^33^ ^]^ UCo_0.8_Ru_0.2_Al^[^ ^34^ ^]^ and Fe_3_Pt.^[^ ^35^ ^]^ These high coefficients are attributed to their non‐zero Berry curvatures at the Fermi level, which contributes significantly to the energy derivative $\frac{\partial \sigma_{zx}}{\partial \varepsilon }{|}_{\varepsilon_{F}}$. Similarly, noncollinear antiferromagnets, such as single‐crystalline Mn_3_Sn^[^ ^36^, ^37^ ^]^ and YbMnBi_2_,^[^ ^38^ ^]^ also display large ANEs. In these materials, non‐zero Berry curvatures are still induced despite low magnetization of canted spins. However, relying solely on such intrinsic material properties to achieve a high *α_zx_* is inherently limited, as other transport properties also significantly affect the transverse thermopower (*S_zx_*). This is evident from the relationship *S_zx_* = *α_xx_ρ_zx_* + *α_zx_ρ_xx_*, where *α_xx_* is the longitudinal TE conductivity, and *ρ_zx_* and *ρ_xx_* are the transverse and longitudinal electrical resistivities, respectively.^[^ ^36^ ^]^ Consequently, developing novel strategies that leverage the tunable parameters of *α_xx_*, *ρ_zx_* and *ρ_xx_* are necessary to further enhance *S_zx_* beyond the intrinsic material properties.

In this study, we employ the following relationship, which shows that the Nernst coefficient (*S*_Nernst_) depends linearly on the Seebeck thermopower (*S*_Seebeck_) and incorporates the contribution of the Hall (or Nernst) angle (*θ*_Hall(Nernst)_) as follows:^[^ ^36^ ^]^

(1) $$S_{\mathrm{Nernst}}=S_{\mathrm{Seebeck}}\;\left(-\tan\theta_{\mathrm{Hall}}+\tan\theta_{\mathrm{Nernst}}\right)$$

where tan θ_Hall_ =   − ρ_*zx*_/ρ_*xx*_ and tan θ_Nernst_ = α_*zx*_/α_*xx*_ . Here, assuming that the ordinary Hall (or Nernst) contribution is negligible and applying the small‐angle approximation, tan θ_Hall(Nernst)_ can be simplified to *θ*_AHE(ANE)_ and it leads to:

(2) $$S_{\mathrm{ANE}}=S_{\mathrm{Seebeck}}\;\left(-\theta_{\mathrm{AHE}}+\theta_{\mathrm{ANE}}\right)$$

where *S*_ANE_ is the anomalous Nernst thermopower and *θ*_AHE(ANE)_ is the anomalous Hall (or Nernst) angle. Given the extensive previous studies on conventional TE materials, which provide numerous methods to enhance *S*_Seebeck_, improving *S*_ANE_ by increasing *S*_Seebeck_ appears to be experimentally feasible. However, to the best of our knowledge, this approach has not been systematically investigated, and efforts to adjust other key parameters such as *θ*_AHE_ and *θ*_ANE_ remain limited.

Meanwhile, disordered‐semiconducting Sr_3_YCo_4_O_11‐δ_ (SYCO) is a promising ANE material, attributed to its ferromagnetic characteristics and a high Seebeck thermopower reaching several hundred µV K^−1^.^[^ ^39^, ^40^, ^41^ ^]^ Notably, applying physical or chemical pressure to SYCO can enhance the Seebeck thermopower by inducing a spin‐state transition in Co ions, which results in a significant entropic contribution to the Seebeck thermopower. In addition, the TE properties of such oxygen‐deficient cobaltates are highly sensitive to oxygen content, as it directly affects electronic transport along Co‐O‐Co pathways. For example, SrCoO_3‐_ *_δ_*, an oxygen non‐stoichiometric compound, exhibits a temperature‐driven sign reversal in its Seebeck thermopower, a phenomenon linked to the effects of oxygen vacancy defects.^[^ ^42^ ^]^ Similarly, layered SrCoO_2.5‐_ *_δ_* thin films with high oxygen deficiency demonstrate an exceptional power factor of 6 mW K^−2^ m^−1^, owing to the synergistic combination of high Seebeck thermopower and low resistivity.^[^ ^43^ ^]^ Furthermore, the potential of the oxygen deficiency to enhance *S*_ANE_ has been reported in epitaxial Fe_4_N/SrTiO_3‐_ *_δ_* films; however, a systematic study on the effects of varying oxygen vacancy concentrations remains absent.^[^ ^44^ ^]^

In this context, we demonstrate a substantial improvement in the *S*_ANE_ of SYCO polycrystals by concurrently enhancing *S*_Seebeck_ and *θ*_ANE_ through the precise control of oxygen‐vacancy defects. Specifically, the introduction of additional oxygen vacancies results in a notable 44% increase in *S*_ANE_, attributed to two key mechanisms: an increased Co^3+^/Co^4+^ concentration ratio caused by the reduction of Co ions, and a decreased Co‐O‐Co bond angle resulting from atomic structural rearrangements. The higher Co^3+^/Co^4+^ ratio increases the total entropy currents of the material, enhancing *S*_Seebeck_, while the reduced bond angle strengthens electron localization, thereby boosting *θ*_ANE_. Combined, these synergistic effects align with predictions from Equation (2), showcasing the potential of oxygen vacancy engineering to optimize ANE materials.

## 2 Results and Discussion

### 2.1 Consistent Physical Structure and Chemical Composition of SYCO Polycrystals Despite Added Co_3_O_4_ Powder

Three distinct SYCO polycrystals were synthesized via a solid‐state reaction, with extra Co_3_O_4_ powder introduced during the powder mixing process to regulate oxygen vacancy levels, as outlined in the Experimental section. To verify the effects of this approach, the microstructures, chemical compositions, crystal structures and atomic structures of the SYCO polycrystals were analyzed using scanning electron microscopy (SEM), inductively coupled plasma (ICP) spectroscopy, X‐ray diffraction (XRD) and scanning transmission electron microscopy (STEM), respectively. These comprehensive analyses confirm the successful introduction of systematically varied oxygen‐vacancy defects into the SYCO samples, resulting in distinct oxygen‐deficient compositions.

**Figure 2:**
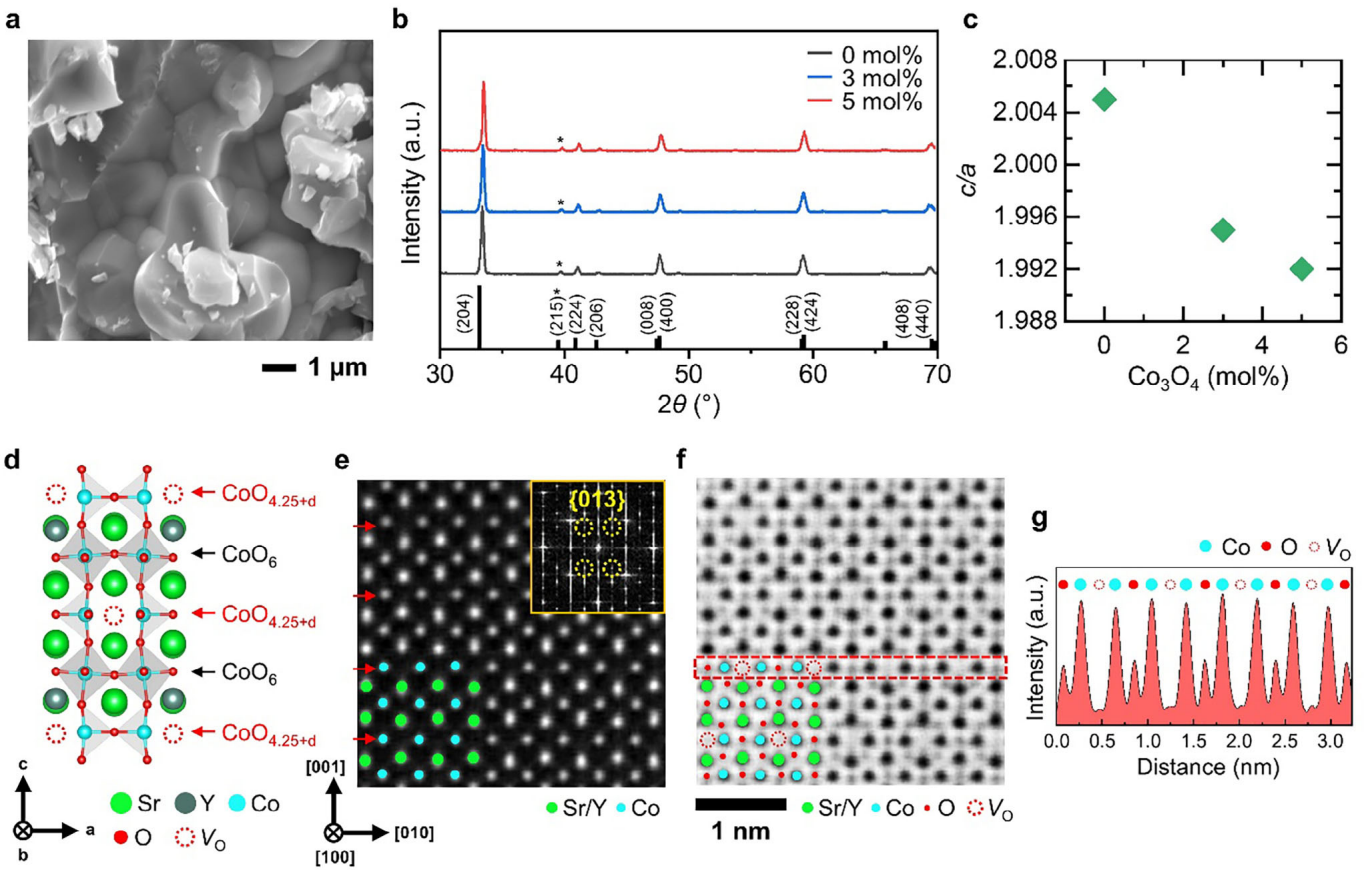
Microstructure and crystal structure analysis of Sr_3_YCo_4_O_11‐δ_ (SYCO) polycrystals. a) SEM image of the SYCO sample synthesized with 0 mol% additional Co_3_O_4_. b) XRD patterns of the SYCO samples synthesized with 0, 3, and 5 mol% additional Co_3_O_4_. Star symbols mark the (215) peak at 2*θ* = 39.5°. c) The relative *c*‐axis lattice constant (*c*/*a*) of the SYCO samples as a function of the added Co_3_O_4_ at room temperature. d) Schematic representation of the SYCO unit cell structure. Light(dark) green spheres represent Sr(Y) atoms, blue spheres correspond to Co atoms, red spheres indicate O atoms, and red dotted spheres denote oxygen vacancies (*V*_O_). e) HAADF‐STEM and f) ABF‐STEM images of the SYCO atomic structures synthesized with 0 mol% additional Co_3_O_4_ along the [100] zone axis. Red arrows highlight the CoO_4.25+d_ layer. The inset in (e) shows the Fast Fourier Transform (FFT) patterns derived from the HAADF image, with yellow dotted circles indicating diffraction spots corresponding to the ordered superstructure of the {013} planes. g) Intensity line profiles extracted from the red dotted box in the ABF‐STEM image shown in (f).

The synthesized SYCO samples exhibit compact structures with micrometer‐sized grains (**Figure** **2**a; Figure S2, Supporting Information), a result of high‐temperature sintering at 1100 °C. These grain sizes are sufficiently large to support the nanometer‐scale range of variable‐range hopping transport, which will be discussed later, and maintain a robust ferrimagnetic phase below the Curie temperature of ≈300 K (Figure 5f). Furthermore, no noticeable secondary phases are detected in the SYCO matrix, eliminating their potential parasitic effects on TE properties.

The ICP analysis reveals that the Sr:Y:Co ratio in all samples remains consistent at 3:1.01 ± 0.05:4.04 ± 0.05, regardless of the addition of extra Co atoms (Table S1, Supporting Information). This suggests that the SYCO polycrystals were synthesized without altering the Co proportion. Instead, the additional Co atoms appear to contribute to increased oxygen vacancy formation, which will be discussed in more detail in a later section. The increase in oxygen vacancies is likely due to the decomposition of Co_3_O_4_ nanopowders into CoO and O during sintering at 1100 °C, as indicated by the Co‐O phase diagram.^[^ ^45^ ^]^ Here, the unstable O atoms likely form O_2_ gas in the ambient atmosphere, creating abundant oxygen vacancies in the lattice. Additionally, a portion of extra Co atoms may have vaporized due to their high vapor pressures,^[^ ^46^ ^]^ which helps preserve the constant Sr:Y:Co ratio of 3:1:4 across all samples.

The XRD analysis (Figure 2b) confirms that all diffraction peaks correspond to the A‐site‐ and oxygen‐vacancy‐ordered structure (*I4/mmm*), characterized by alternately stacked O‐rich octahedral CoO_6_ layers and O‐deficient tetrahedral CoO_4.25+d_ (d: the amount of excess oxygen) layers along the *c*‐axis (Figure 2d; Figure S1, Supporting Information). Notably, no secondary phases are observed, and the presence of (215) peak at 2*θ* = 39.5° indicates an ordered superstructure consistent with the tetragonal extinction law.^[^ ^47^ ^]^ Moreover, the XRD peaks exhibit a strong agreement with the simulated patterns obtained from Rietveld refinement, further confirming that the SYCO samples adopt an ordered tetragonal phase (Figure S3, Supporting Information). However, with increasing Co_3_O_4_, the overall peaks shift to higher 2*θ* values (Figure S4, Supporting Information), indicating a reduction in the lattice parameter. This, in turn, results in a 0.6% decrease in the relative *c*‐axis lattice constant (*c*/*a*) from 2.005 to 1.992 (Figure 2c; Table S2, Supporting Information), which will be discussed in detail in a later section.

The high‐angle annular dark‐field (HAADF) and annular bright‐field (ABF) STEM images (Figure 2e,f; Figure S5, Supporting Information) further confirm the oxygen‐vacancy‐ordered structures in both samples synthesized with 0 and 5 mol% additional Co_3_O_4_. In these images, the intensity of individual atoms is approximately proportional to the square of their atomic number. For clarity, Sr/Y, Co and O atoms are indicated by green, blue, and red circles, respectively, while oxygen vacancies (*V*_O_) are represented as red dotted circles. Notably, the superlattice {013} diffractions spots observed in the Fast Fourier Transform (FFT) patterns (inset of Figure 2e) corroborate the presence of ordered superstructures, which align well with the XRD findings. Furthermore, the intensity line profile (Figure 2g), extracted from the red dotted box in the ABF‐STEM image in Figure 2f, exhibits an alternating intensity values corresponding to O, Co and *V*_O_ along the [010] direction (parallel to the *a*‐axis). This pattern highlights the predominant localization of oxygen vacancies within the CoO_4.25+d_ layers.^[^ ^48^ ^]^ Therefore, it can be concluded that the oxygen‐vacancy‐ordered structures remain consistent, regardless of the amount of additional Co_3_O_4_.

### 2.2 The Generation of Oxygen Vacancies and the Associated Reduction of Co Ions

**Figure 3:**
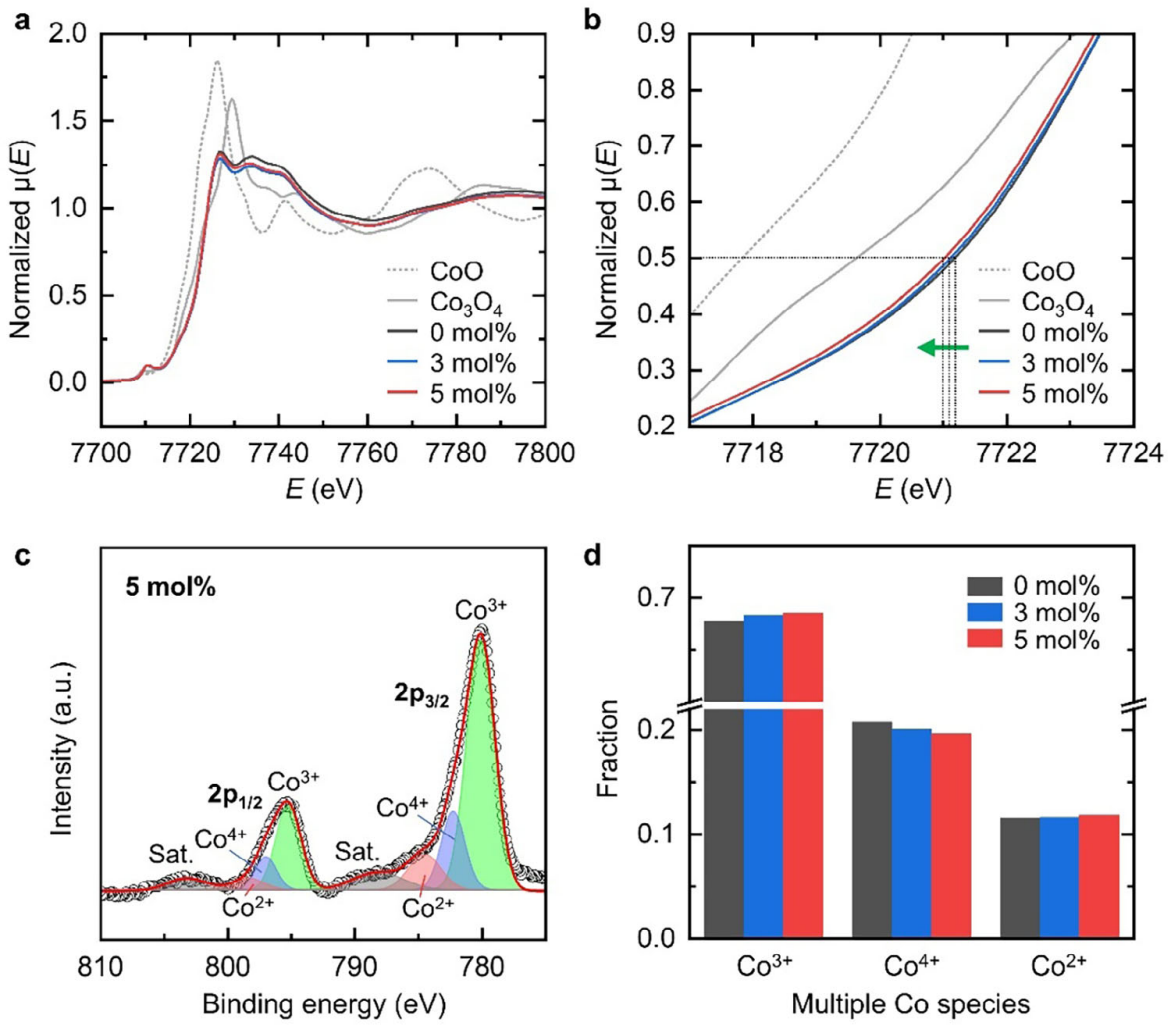
Analysis of Co reduction in the SYCO polycrystals. a) The normalized Co K‐edge XANES spectra of SYCO samples compared with reference Co oxides, including CoO and Co_3_O_4_. b) Enlarged view of (a) in the energy (*E*) range from 7717 to 7724 eV at the μ = 0.5 edge step. c) The Co‐2p XPS spectra of SYCO sample with 5 mol% additional Co_3_O_4_, along with peak fitting results based on mixed‐valence Co ions. d) Estimated fraction of Co species (Co^2+^, Co^3+^ and Co^4+^ ions) for each sample.

The oxygen vacancy concentration generated in each SYCO polycrystal, and the corresponding reduction of Co ions were analyzed using X‐ray absorption near‐edge structure (XANES) spectroscopy. Specifically, normalized Co K‐edge XANES spectra (**Figure** **3**a), featuring characteristic main peaks at ≈7726 eV, were employed to estimate Co oxidation states.^[^ ^49^, ^50^, ^51^ ^]^ In more detail, the absorption energies at μ = 0.5 (*E*_μ = 0.5_) were determined as the main peak energies for each sample using the half‐height method (Figure 3b).^[^ ^52^ ^]^ The oxidation states were then extrapolated by comparison with reference Co oxides, namely 2+ for CoO and 2.67+ for Co_3_O_4_, as summarized in **Table** **1**. The estimated Co valences for the SYCO samples with 0, 3, and 5 mol% of extra Co_3_O_4_ were determined to be 3.24+, 3.21+, and 3.18+, respectively. These Co valences correspond to oxygen vacancy concentrations of *δ* = 0.02, 0.08 and 0.14, in good agreement with previously reported values.^[^ ^53^ ^]^ Moreover, X‐ray photoelectron spectroscopy (XPS) analysis (Figure 3c; Figure S7, Supporting Information), where the Co‐2p spectra were deconvoluted into peaks corresponding to mixed‐valence Co ions (Co^2+^, Co^3+^ and Co^4+^),^[^ ^54^, ^55^ ^]^ reveals a qualitative distribution among multiple Co species. As shown in Figure 3d, with a negligible fraction of Co^2+^ ions, Co^3+^ ions dominate the mixed‐valence state, while the remaining fraction consists of Co^4+^ ions. Thus, it can be concluded that the relative proportion *p* of Co^4+^ ions compared to Co^3+^ decreases with increasing *δ* (Table 3), reflecting the partial reduction of Co^4+^ ions to Co^3+^ ions to maintain charge neutrality.

**Table 1:** The absorption energies at μ = 0.5 (*E*_μ = 0.5_) and the estimated oxidation states of Co ions in the SYCO samples, along with reference Co oxides such as CoO and Co_3_O_4_.

	*E*_μ = 0.5_ [eV]	Oxidation state of Co ions
CoO	7717.83	2
Co_3_O_4_	7719.63	2.67
0 mol%	7721.18	3.24
3 mol%	7721.11	3.21
5 mol%	7721.02	3.18

Therefore, it is confirmed that the addition of extra Co_3_O_4_ powders successfully introduced oxygen‐vacancy defects into the SYCO samples, resulting in varying levels of oxygen deficiency. These systematically prepared SYCO polycrystals are anticipated to serve as an effective experimental platform for investigating changes in ANE performance, as a function of oxygen vacancy concentration.

### 2.3 The Impact of Oxygen Vacancies on the Octahedral Structure of SYCO

**Figure 4:**
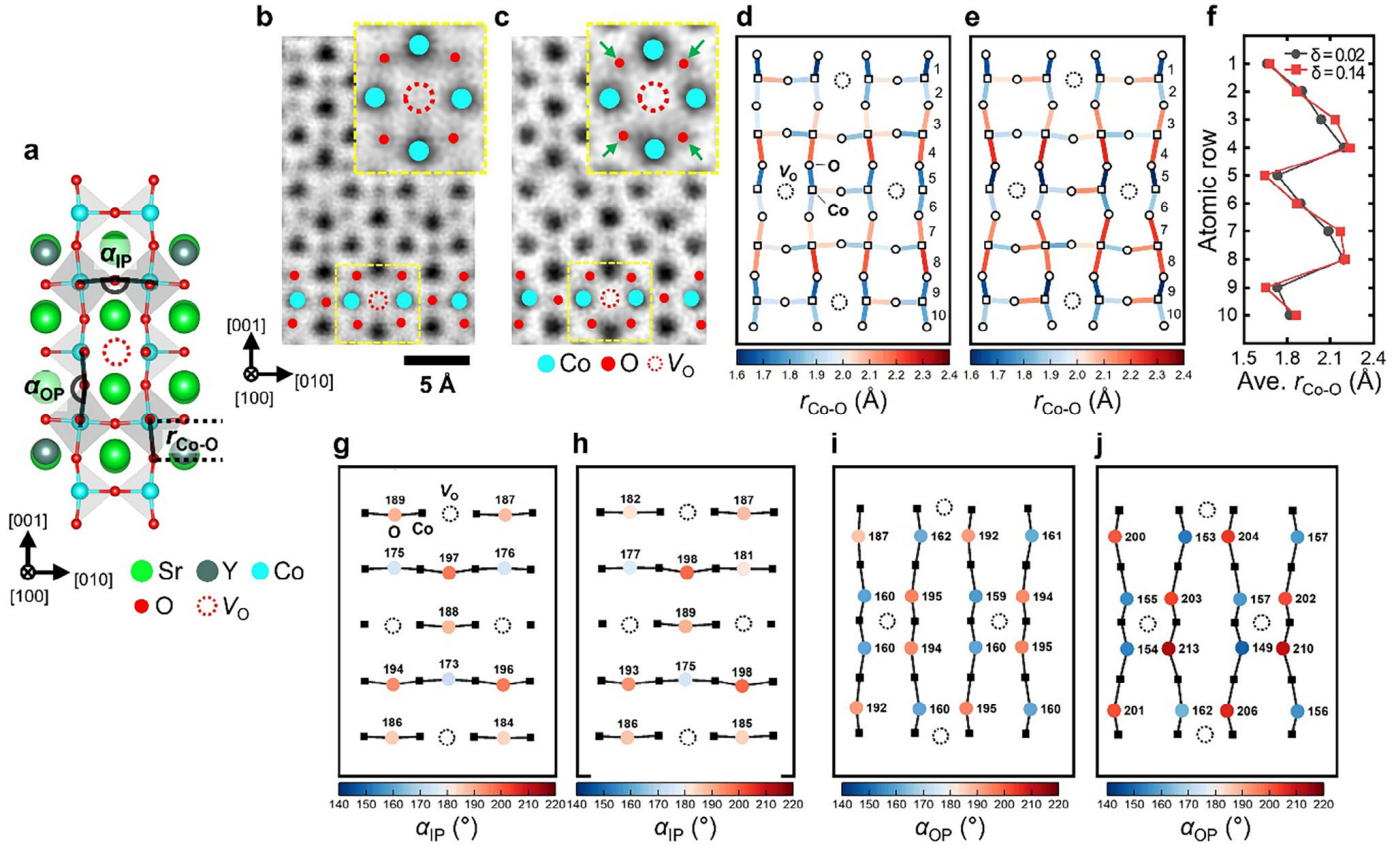
ABF‐STEM image analysis of the octahedral structures in the SYCO samples with *δ* = 0.02 and *δ* = 0.14. a) Schematic representation of Co‐O bond lengths and Co‐O‐Co bond angles within the SYCO unit cell, where *r*_Co‐O_ denotes the Co‐O bond length, and *α*_IP_/*α*_OP_ indicates the in‐plane/out‐of‐plane Co‐O‐Co bond angles, respectively. b,c) ABF‐STEM images along the [100] zone axis for samples with (b) *δ* = 0.02 and (c) *δ* = 0.14. The insets show enlarged regions near *V*_O_, highlighted by yellow dotted boxes in the ABF‐STEM images. d,e) *r*_Co‐O_ mappings for (d) *δ* = 0.02 and (e) *δ* = 0.14. f) Average out‐of‐plane *r*_Co‐O_ measurements for each atomic row in both samples. g,h) *α*_IP_ and i,j) *α*_OP_ mappings for (g,i) *δ* = 0.02 and (h,j) *δ* = 0.14.

Meanwhile, previous studies^[^ ^56^, ^57^ ^]^ suggest that such variations in oxygen vacancy concentration are highly likely to induce structural distortions in the octahedral configurations formed by central Co atoms and their neighboring O atoms (**Figure** **4**a). To investigate these changes, we analyzed the arrangements of Co, O and *V*_O_ in samples with *δ* = 0.02 and *δ* = 0.14, using data extracted from ABF‐STEM images (Figure 4b,c). The resulting arrangements are visually represented in Figure 4d,e, where distinct symbols are used: blank squares indicate Co atoms, blank circles represent O atoms, and black dotted circles denote oxygen vacancies. From this analysis, we measured the Co‐O bond lengths and the Co‐O‐Co bond angles for each sample, with their variations illustrated using a color gradient ranging from dark blue (indicating lower values) to dark red (indicating higher value). The Co‐O bond length (*r*_Co‐O_) along the [010] direction (parallel to the *a*‐axis) shows minimal variation between the two samples, with average values of 1.95 ± 0.05 Å for *δ* = 0.02 and 2.00 ± 0.05 Å for *δ* = 0.14. In contrast, the *r*_Co‐O_ along the [001] direction (parallel to the *c*‐axis) exhibits significant variations for *δ* = 0.14, as evidenced by a more pronounced color contrast compared to *δ* = 0.02. This behavior arises because, at higher oxygen vacancy centration (*δ* = 0.14), oxygen atoms closest to the vacancies shift further inward to maintain charge neutrality, as indicated by the green arrows in the inset of Figure 4c. Consequently, as shown in Figure 4f, the average out‐of‐plane *r*_Co‐O_ for *δ* = 0.14 alternates between shorter distances (e.g., rows 5 and 6, which are closer to oxygen vacancies) and longer distances (e.g., rows 4 and 7, which are farther from oxygen vacancies) compared to *δ* = 0.02. In terms of the bond angles, the in‐plane Co‐O‐Co bond angle (*α*_IP_; Figure 4g,h) remains nearly constant regardless of the oxygen vacancy concentration, with average values of 186° for both samples. However, the out‐of‐plane Co‐O‐Co bond angle (*α*_OP_; Figure 4i,j) deviates more significantly from 180° for *δ* = 0.14 compared to *δ* = 0.02, with average deviations of 16.4 ± 0.5° for *δ* = 0.02 and 24.8 ± 0.5° for *δ* = 0.14. These out‐of‐plane structural distortions within the octahedral configuration seem to induce compressive strain along the *c*‐axis, leading to a global reduction in the relative *c*‐axis lattice constant (*c*/*a*), as observed in the XRD findings. Such distortions likely play a critical role in modulating electronic transport along Co‐O‐Co pathways and may significantly influence TE properties, as discussed in subsequent sections.

### 2.4 Enhancement of Anomalous Nernst Thermopower with High Oxygen Vacancy Concentration

**Figure 5:**
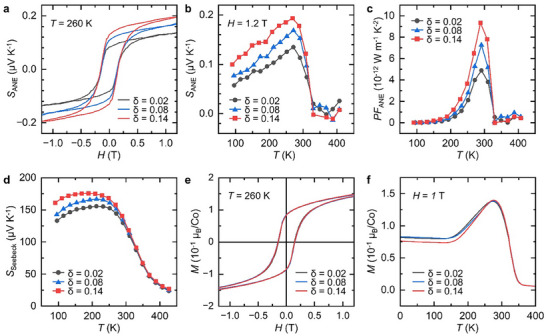
Thermoelectric and magnetic properties of SYCO samples as a function of oxygen vacancy concentration (*δ*). a) *H* dependence of the anomalous Nernst thermopower (*S*_ANE_) at 260 K. b) *T* dependence of *S*_ANE_ at 1.2 T. c) *T* dependence of the ANE‐based power factor (*PF*_ANE_). d) *T* dependence of the Seebeck thermopower (*S*_Seebeck_). e) *H* dependence of the magnetic moment (*M*) at 260 K. f) *T* dependence of *M* under field cooling at *H* = 1 T.

We now examine the behavior of *S*_ANE_ in the SYCO polycrystals as a function of *δ* variation. As shown in the *H* dependence of *S*_ANE_ at 260 K (**Figure** **5**a), *S*_ANE_ exhibits the hysteresis loops and saturates at higher magnetic fields, similar to the *H* dependence of the magnetic moment (*M*) shown in Figure 5e. Furthermore, as shown in Figure 5b, *S*_ANE_ at 1.2 T gradually increases with temperature (*T*), reaching a peak at 260 K before rapidly declining to nearly zero at higher temperatures. In cobaltate‐based materials, the ANE originates from ferromagnetic long‐range ordering.^[^ ^58^, ^59^ ^]^ Therefore, it is unsurprising that the *S*_ANE_‐*H* and *S*_ANE_‐*T* curves align with the ferromagnetic behavior of the *M* for each sample, as shown in Figure 5e,f. In particular, the changing trend in the *H* dependence of *S*_ANE_—where *S*_ANE_ increases with *H* and then saturates—arises because a higher *M* generates a stronger anomalous Nernst field, consistent with the scaling relation.^[^ ^60^ ^]^ Interestingly, the maximum *S*_ANE_ at 260 K increases significantly with *δ*. For example, the *S*_ANE_ of the *δ* = 0.14 sample is 44% higher than that of the *δ* = 0.02 sample. Accordingly, the ANE‐based power factor (*PF*_ANE_ = *S*_ANE_^2^/*ρ_xx_*), where *ρ_xx_* is provided in Figure S8, Supporting Information, also reaches its maximum, mirroring the behavior of *S*_ANE_; notably, the *PF*_ANE_ of the *δ* = 0.14 sample is 91.2% higher than that of the *δ* = 0.02 sample (Figure 5c). According to the literature, *S*_ANE_ often scales proportionally with the magnitude of *M* in ferromagnets.^[^ ^60^ ^]^ However, no significant variation in *M* was observed among the SYCO samples at 260 K across different *δ* values (Figure 5e). This suggests that factors other than *M* contribute to the observed increase in *S*_ANE_ via a different mechanism. To identify these factors, three parameters—*S*_Seebeck_, *θ*_AHE_ and *θ*_ANE_—were investigated based on the relationship defined in Equation (2), where *S*_ANE_ is expressed as the product of *S*_Seebeck_ and (‐*θ*_AHE_ + *θ*_ANE_). First, *S*_Seebeck_ is positive at all temperatures, indicating hole‐type majority charge carriers originating from Co^4+^ ions (Figure 5d). Additionally, *S*_Seebeck_ gradually increases with *T* but sharply decreases above 300 K, similar to the behavior of *S*_ANE_, as both are influenced by the ferromagnetic‐to‐paramagnetic transition (Figure 5f). This transition disrupts the spin‐configuration entropy of Co ions, an essential factor in maintaining high Seebeck values as described in Equation (3), ultimately resulting in a negligibly small *S*_Seebeck_ above 300 K. A more detailed discussion of this mechanism is provided in a later section. Moreover, *S*_Seebeck_ increases gradually with *δ*, reaching 158 µV K^−1^ for *δ* = 0.14 at 260 K, which represents a 9.7% increase compared to *δ* = 0.02. Meanwhile, the sum of ‐*θ*_AHE_ and *θ*_ANE_ at 260 K, calculated using the measured *S*_Seebeck_, *S*_ANE_, *ρ_xx_* and *ρ_zx_* (See Figures S8 and S9, Supporting Information for details), also shows a significant increase with *δ* (**Table** **2**), and this rise is primarily driven by a substantial 31% rise in *θ*_ANE_, accompanied by a negligible reduction in |*θ*_AHE_|. Consequently, the combined increases in *S*_Seebeck_ and *θ*_ANE_ result in a 44% enhancement of *S*_ANE_ in the sample with the highest oxygen vacancy concentration (*δ* = 0.14), suggesting a critical role of oxygen vacancies in enhancing *S*_ANE_.

**Table 2:** The anomalous Hall angle (*θ*_AHE_) and anomalous Nernst angle (*θ*_ANE_) at 260 K, along with the sum of ‐*θ*_AHE_ and *θ*_ANE_ for samples with *δ* = 0.02, 0.08, and 0.14.

*δ*	*θ*_AHE_	*θ*_ANE_	‐*θ*_AHE_ + *θ*_ANE_
0.02	−1.561·10^−4^	7.773·10^−4^	9.334·10^−4^
0.08	−1.269·10^−4^	9.891·10^−4^	1.116·10^−3^
0.14	−1.273·10^−4^	1.097·10^−3^	1.224·10^−3^

### 2.5 Discussion on the Enhancement of the Seebeck Thermopower in SYCO Samples

With the *S*_ANE_ significantly enhanced by increasing *δ—*attributed to the improvements in both *S*_Seebeck_ and *θ*_ANE_ *—*it is now imperative to systematically investigate the specific effects of oxygen vacancy formation on *S*_Seebeck_ and *θ*_ANE_.

First, the *S*_Seebeck_ slightly decreases as *T* decreases for all samples, exhibiting a positive curvature (Figure 5c), which is characteristic of disordered semiconductors.^[^ ^41^ ^]^ The Seebeeck effect in such disordered semiconductors is commonly described empirically using the extended Heikes formula:^[^ ^61^, ^62^ ^]^

(3) $$\begin{array}{c} S_{\mathrm{Heikes}}=-\frac{k_{B}}{e}\;\ln\left[\frac{g_{3}}{g_{4}}\left(\frac{p}{1-p}\right)\right]=\frac{k_{B}}{e}\;\ln\left(\frac{g_{4}}{g_{3}}\right)+\frac{k_{B}}{e}\ln\left(\frac{1-p}{p}\right) \end{array}$$

where *S*_Heikes_ represents the Seebeck thermopower based on the Heikes model, *g*_3_ and *g*_4_ are the spin‐orbital degeneracies of Co^3+^ and Co^4+^, respectively, *p* denotes Co^4+^ concentration and *k*_B_ is the Boltzmann constant. Here, the Seebeck thermopower corresponds to the entropy per hole carrier generated by the minority Co^4+^ ions within a Co^3+^ ion background.

The Heikes thermopower can be expressed as the sum of two components, $S_{1}=\frac{k_{B}}{e}\;\ln\left(\frac{g_{4}}{g_{3}}\right)$ and $S_{2}=\frac{k_{B}}{e}\;\ln\left(\frac{1-p}{p}\right)$. The first term, *S*_1_, represents the Seebeck voltage resulting from the spin configuration entropy of Co^3+^ and Co^4+^ ions, and it increases as the spin‐orbital degeneracy ratio *g*_4_/*g*_3_ increases. Notably, this ratio depends on the spin states of Co^3+^ and Co^4+^ ions (Figure S10, Supporting Information)^[^ ^61^ ^]^ and can be approximately determined by analyzing the temperature‐dependent magnetization. As shown in Figure 5f, the *M*‐*T* curves remain flat at very low temperatures below 150 K, then abruptly increase with temperature, reaching maximum points ≈260 K. The sudden increase in *M* with *T* suggests that that most Co ions adopt low‐spin (LS) ground states, while a significant fraction of LS‐Co^3+^ and LS‐Co^4+^ ions are thermally activated to higher spin states at elevated temperatures. Based on these observations, *g*_3_ and *g*_4_ for all samples can be roughly estimated as 1 for LS‐Co^3+^ and 6 for LS‐Co^4+^, respectively, resulting in *g*_4_/*g*_3_ = 6 and *S*_1_ = (*k*_B_ ln(6))/*e* = 150 µV K^−1^.^[^ ^61^ ^]^ Interestingly, this calculated value aligns closely with the experimental *S*_Seebeck_ for the *δ* = 0.02 sample, which reaches ≈150 µV K^−1^ at 260 K. Therefore, it can be concluded that the Seebeck voltage prominently originates from strong spin entropy differences between Co^3+^ and Co^4+^ ions, driven by their low‐spin‐state characteristics.

The second term, *S*_2_, represents the Seebeck voltage generated by the entropy associated with the occupation of the spin states on Co^3+^ and Co^4+^ ions. Using the relative proportion of Co^4+^ ions to Co^3+^ ions (*p*) (**Table** **3**), as determined from the XPS and XANES analyses, $\frac{1-p}{p}$ can be calculated, allowing subsequent estimation of *S*_2_. As summarized in Table 3, $\frac{1-p}{p}$ increases with *δ* due to the partial conversion of Co^4+^ ions into Co^3+^ ions caused by the introduction of additional oxygen vacancies. This, in turn, leads to a concurrent increase in *S*_2_, given the relationship of $S_{2}=\frac{k_{B}}{e}\;\ln\left(\frac{1-p}{p}\right)$. Although the estimated *S*_2_ (a few hundred µV K^−1^) appears quite high, most likely due to the qualitative estimation of *p*, the variation in *S*_2_ among the samples remains significant. For example, *S*_2_ for the *δ* = 0.14 sample is 30% higher than that of the *δ* = 0.02 sample, which qualitatively aligns with the 9.7% increase in the experimental *S*_Seebeck_, as shown in Figure 5c. These results suggest that the slight reduction of Co^4+^ ions, and the resulting increase in mixed valency, enhance the entropy‐driven charge transport in the SYCO system, thereby improving *S*_Seebeck_ by several µV K^−1^.

**Table 3:** The relative proportion of Co^4+^ ions to Co^3+^ ions (*p*) and the calculated second term of the Heikes thermopower (*S*_2_) for samples with *δ* = 0.02, 0.08, and 0.14.

*δ*	*p*	(1‐*p*)/*p*	*S*_2_ [µV K^−1^]
0.02	0.238	3.20	100
0.08	0.214	3.67	112
0.14	0.180	4.56	131

### 2.6 Discussion on the Enhancement of the Anomalous Nernst Angle in SYCO Samples

Not only does the increase in *S*_Seebeck_ play a significant role in enhancing *S*_ANE_, but *θ*_ANE_ also contributes substantially, as described in Equation (2). Specifically, *θ*_ANE_ is defined as *θ*_ANE_ = *α_zx_*/*α_xx_*, and the Mott relation, $\alpha_{xx(zx)}=\frac{\pi^{2}{k_{B}}^{2}}{3e}\;T\frac{\partial \sigma_{xx(zx)}}{\partial \varepsilon }{|}_{\varepsilon_{F}}$ (*σ_xx_*: longitudinal electrical conductivity), holds for the disordered SYCO system because both the anomalous Hall effect and the anomalous Nernst effect share a common physical origin.^[^ ^63^, ^64^, ^65^, ^66^ ^]^ Given this, changes in electrical transport properties, particularly in *σ_xx_* and *σ_zx_*, are likely critical for the observed enhancement in *θ*_ANE_.

**Figure 6:**
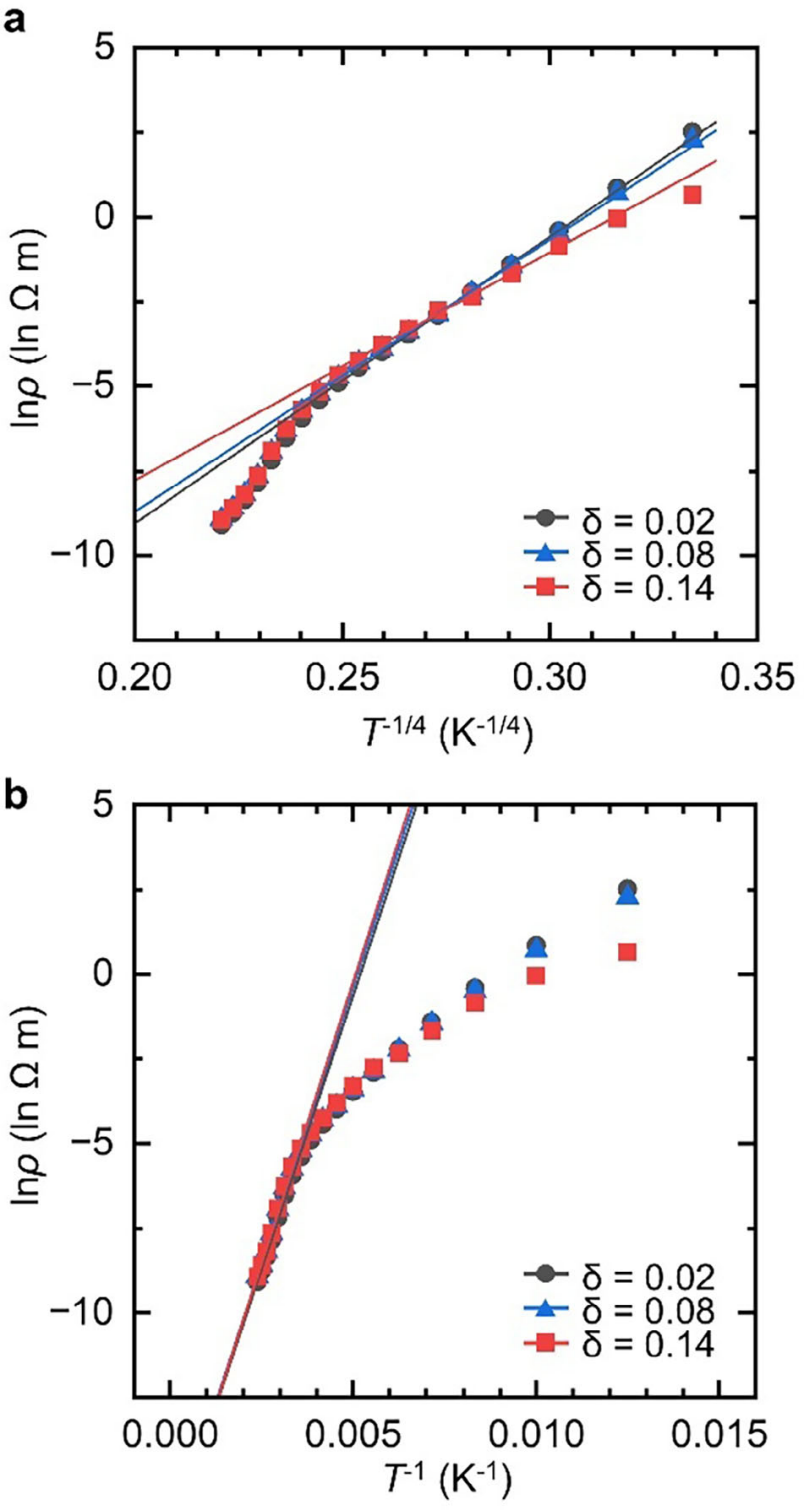
The electrical transport properties of the SYCO polycrystals. The ln(*ρ*) plotted as a function of *T* ^−1/4^ in (a) and *T* ^−1^ in (b) for samples with *δ* = 0.02, 0.08 and 0.14. The straight lines represent the fittings for the variable‐range hopping (VRH) regime in (a), spanning 100 to 240 K, and the Arrhenius regime in (b), observed from 280 K to 420 K.

**Figure 7:**
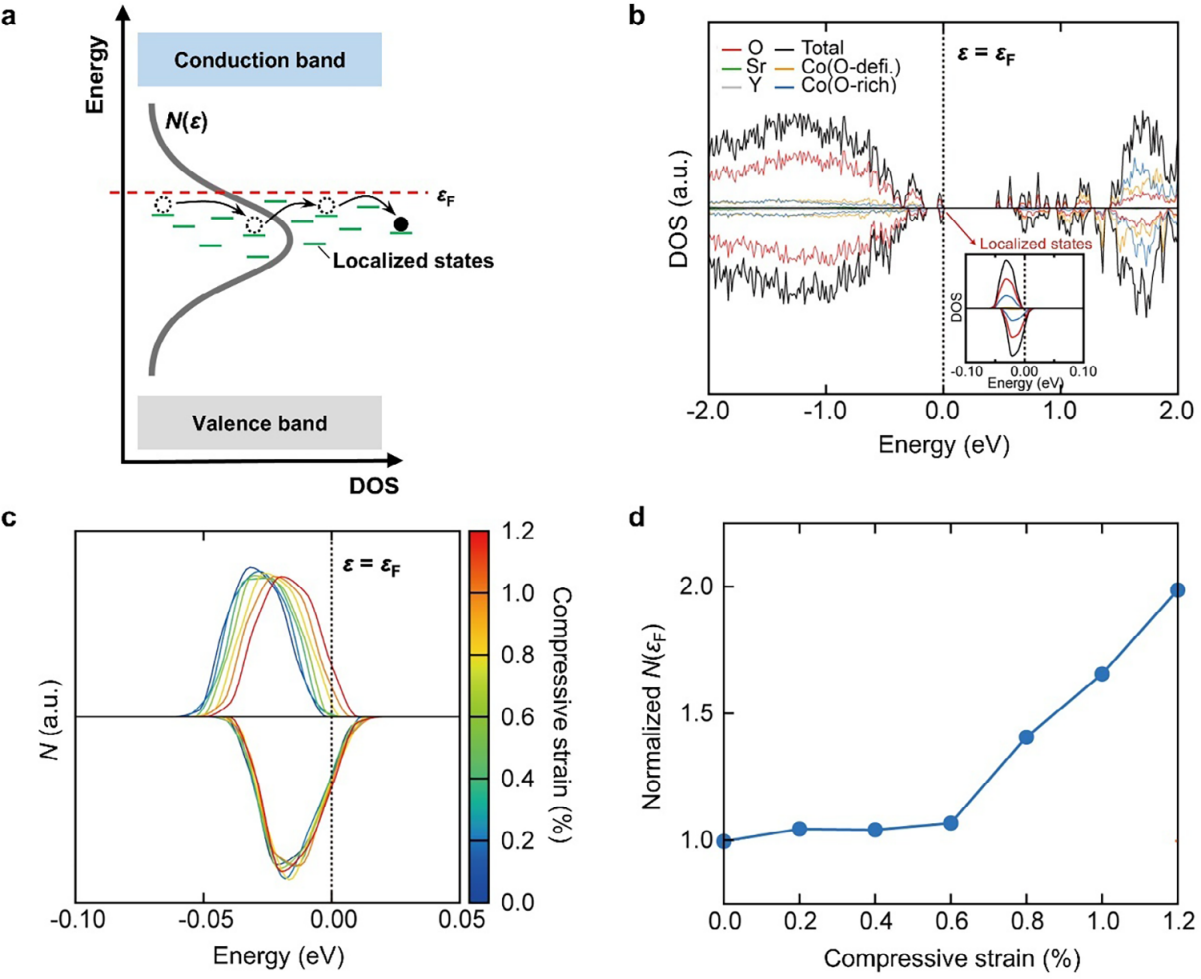
Theoretical approach for variable‐range hopping (VRH) using DFT calculations for the most stable structure of Sr_3_YCo_4_O_11_. a) Schematic representation of VRH conduction via localized states near the Fermi level (*ε*_F_). b) The total and projected density of states (DOS), with the inset highlighting localized states near *ε*_F_. c) The evolution of local DOS (*N*) near *ε*_F_ with increasing compressive strain. d) Normalized total *N*(*ε*_F_) relative to the unstrained case as a function of compressive strain.

As shown in **Figure** **6**, the electrical conduction behavior of the disordered semiconducting SYCO system can be effectively analyzed by examining the *T* dependence of electrical resistivity *ρ*. At low temperatures, conduction is governed by the Mott variable‐range hopping (VRH) mechanism (Figure 6a), whereas at high temperatures, it transitions to an activated Arrhenius‐type mechanism (Figure 6b). In particular, at low temperatures (≤ 260 K), where the ANE is observed, ln *ρ* varies linearly with *T* ^−1/4^, consistent with the Mott VRH model:^[^ ^67^, ^68^, ^69^ ^]^

(4) $$\rho /\rho_{0}=\exp{\left(T_{0}/T\right)}^{1/4}$$

where *ρ*_0_ is a prefactor, and *T*_0_ is the Mott characteristic temperature. The values of *T*_0_, which represent the hopping energy required to activate localized electrons, are obtained from fitting the data (**Table** **4**). The VRH model describes charge transport as hopping between randomly distributed localized electronic states, distinct from the continuous band conduction mechanism of conventional semiconductors. In the SYCO system, incoherent hopping conduction occurs within the O‐rich CoO_6_ layers, where hole carriers jump between localized states near the Fermi level (**Figure** **7**a).^[^ ^41^, ^47^ ^]^

**Table 4:** The Mott characteristic temperature (*T*_0_) fitted to the low‐temperature *ρ*, the Arrhenius activation energy (*E*_a_) fitted to the high‐temperature *ρ*, and the estimated local DOS near the Fermi level (*N*(*ε*_F_)) as functions of the oxygen vacancy concentration (*δ*).

*δ*	*T*_0_ [K]	*E*_a_ [eV]	*N*(*ε*_F_) [×10^20^ eV^−1^ cm^−3^]
0.02	5.126 × 10^7^	0.2749	6.412
0.08	4.159× 10^7^	0.2822	7.903
0.14	2.053× 10^7^	0.2878	16.01

Under the VRH conduction, *σ_xx_* and *σ_zx_* can be expressed as:^[^ ^63^ ^]^

(5) $$\sigma_{xx}/\sigma_{xx,0}=\exp\left[-{\left(T_{0}/T\right)}^{1/4}\right]$$

(6) $$\sigma_{zx}/\sigma_{zx,0}={\sigma_{xx}}^{2}\;{\left.\frac{d\ln N}{d\varepsilon }\right|}_{\varepsilon_{F}}\frac{hT}{e^{2}}{\left(T_{0}/T\right)}^{1/4}\exp\left[-{\left(T_{0}/T\right)}^{1/4}\right]$$

where *σ_xx_*_,0_ and *σ_zx_*_,0_ are prefactors, and *N* represents the local density of states (DOS). Substituting these expressions into $\theta_{\mathrm{ANE}}=\frac{{\partial \sigma_{zx}/\partial \varepsilon |}_{\varepsilon_{F}}}{{\partial \sigma_{xx}/\partial \varepsilon |}_{\varepsilon_{F}}}\;$ yields:

(7) $$\theta_{\mathrm{ANE}}∼\exp\left[-2{\left(T_{0}/T_{F}\right)}^{1/4}\right]\left[{\left(T_{0}/T_{F}\right)}^{1/4}+1\right]{\left.\frac{d\ln N}{d\varepsilon }\right|}_{\varepsilon_{F}}$$

where *T*_F_ is the Fermi temperature. The subsequent analysis focuses on the components *T*_0_ and *N* to elucidate the mechanisms underlying the enhancement of *θ*_ANE_ observed in the SYCO samples.

First, the fitted *T*_0_ values for the SYCO polycrystals decrease with increasing *δ* (Table 4), indicating an increased hopping probability with the introduction of additional oxygen vacancies. Specifically, *T*_0_ is inversely proportional to both the localization length (*f*_0_) and the local DOS near the Fermi level (*N*(*ε*_F_)):

(8) $$T_{0}=\beta /\left(k_{B}{f_{0}}^{3}N\left(\varepsilon_{F}\right)\right)$$

where *β* is a numerical factor ≈ 21.^[^ ^69^, ^70^ ^]^ Thus, increases in either *f*_0_ or *N*(*ε*_F_) could lead to a decrease in *T*_0_. Changes in *f*_0_ and *N*(*ε*_F_) are closely tied to the structural distortions previously observed via STEM (Figure 4): the presence of oxygen vacancies induces the local compressive strain along the *c*‐axis by altering O‐atom positions to maintain charge neutrality, causing the buckling in the Co‐O‐Co bond angle. In here, if *f*_0_ corresponds to the Co‐O bond length in the CoO_6_ layers—where the VRH conduction occurs through Co ions^[^ ^69^, ^71^ ^]^—the largely preserved in‐plane atomic structure ensures *f*_0_ remains unchanged despite the presence of additional oxygen vacancies. For example, *f*_0_ is estimated to be 1.95 Å by averaging the in‐plane *r*_Co‐O_, particularly in the CoO_6_ layers. In contrast, *N*(*ε*_F_) is influenced by changes in the electronic band structure driven by the atomic rearrangements. Notably, first‐principles density functional theory (DFT) calculations (Figure 7b–d) suggest that *c*‐axis compressive strain plays a key role in the evolution of *N*(*ε*_F_).

As illustrated in Figure 7b and Figure S11, Supporting Information, the most stable structure of Sr_3_YCo_4_O_11_ features localized states near the Fermi level, primarily originating from the O‐rich CoO_6_ layers (indicated by blue lines in Figure 7b). In this ground‐state configuration, spin‐polarized splitting occurs, with fully occupied up‐spin bands and partially filled down‐spin bands. Notably, under compressive strain along the *c*‐axis, the local DOS—particularly for the up‐spin bands—shifts to higher energies, whereas the down‐spin bands remain largely unaffected. This, in turn, leads to an increase in the total *N*(*ε*_F_), which represents the sum of the local DOS for the up‐spin and down‐spin bands (Figure 7c). Consequently, the enhancement in *N*(*ε*_F_) lowers *T*_0_, as described by Equation (8), consistent with previous findings that report a similar trend under applied physical pressure.^[^ ^41^ ^]^ Moreover, with increasing the compressive strain, the additional partial occupation of the up‐spin bands at the Fermi level further accelerates the increase in *N*(*ε*_F_) (Figure 7d). This behavior qualitatively aligns with experimentally derived *N*(*ε*_F_) using Equation 8, based on estimated *f*_0_ and *T*_0_ (Table 4). Initially, *N*(*ε*_F_) increases gradually as *δ* varies from 0.02 to 0.08, followed by a sharp upturn near *δ* = 0.14.

The enhancement of *N*(*ε*_F_) due to the increased compressive strain is closely linked to variations in crystal‐field splitting (Δ_CF_) within Co 3*d* orbitals.^[^ ^71^, ^72^, ^73^, ^74^ ^]^ Specifically, Δ_CF_ increases under compression, and when it exceeds the Hund exchange energy, it promotes the localization of lower‐energy electrons, thereby stabilizing lower‐spin states (Figure S10, Supporting Information). This mechanism effectively explains the magnetic behavior observed in our SYCO samples, where *M* at low temperatures slightly decreases with increasing *δ* (Figure 5f), as well as the reduced spin polarization in the DFT results. Moreover, since Δ_CF_ is generally correlated with the band gap—approximately twice the thermal activation energy (*E*_a_)—the observed increase in *E*_a_ with *δ* (Table 4) further supports the strengthening of Δ_CF_. Here, *E*_a_ is determined from high‐temperature fitting based on the relationship ρ/ρ_0_ = exp (*E*_a_/*k*_B_ *T*)  shown in Figure 6b.

Now we can interpret the observed increase in *θ*_ANE_ for the SYCO sample with the highest oxygen vacancy concentration (*δ* = 0.14) by considering the atomic structural distortions. First, the constant *f*_0_, derived from the stable atomic arrangements within the CoO_6_ layers, ensures that the hopping distance across the localized states remain robust, even with the introduction of oxygen vacancies. Second, the simultaneous increase in *N*(*ε*_F_), caused by the enhanced electron localization on Co ions, establishes a VRH‐preferrable environment at low temperatures. Consequently, the combination of unchanged *f*_0_ and increased *N*(*ε*_F_) results in a lower *T*_0_, which, in turn, contributes to the rise in *θ*_ANE_ through the monotonically increasing function *f* (*T*_0_) =  *exp*[− 2(*T*_0_/*T_F_*)^1/4^][(*T*_0_/*T_F_*)^1/4^ + 1] with decreasing *T*_0_ in Equation (7) (Figure S12, Supporting Information). This substantial improvement in *S*_ANE_ of the SYCO polycrystals, achieved by concurrently enhancing *S*_Seebeck_ and *θ*_ANE_, provides a guideline for developing efficient ANE materials through extrinsic methods. This approach also broadens the scope of ANE materials beyond topological systems, paving the way for future transverse TE applications. Furthermore, SYCO polycrystals offer strong potential for room‐temperature TE applications, including maintenance‐free energy harvesting and solid‐state cooling for IoT devices, with additional potential in body‐heat‐powered wearable devices, while utilizing the stability of oxide‐based TE materials.

## 3 Conclusion

We present a facile strategy for enhancing *S*_ANE_, a transverse TE performance parameter, by introducing additional oxygen vacancies into SYCO polycrystals. The slight reduction in Co^4+^ ions with increasing *δ* enhances the mixed valency of Co ions, driving higher entropy‐driven currents and improving *S*_Seebeck_. Simultaneously, *c*‐axis compressive strain, induced by the buckling of the Co‐O‐Co bond angle, increases the local DOS near the Fermi level, further enhancing *θ*_ANE_. The improvement in both *S*_Seebeck_ and *θ*_ANE_ leads to a substantial enhancement of *S*_ANE_. Our findings demonstrate a viable approach to improving ANE performance by tuning oxygen vacancies—an extrinsic factor—rather than relying solely on the intrinsic material properties. This approach not only provides valuable insights into extrinsic ANE control but also expands the range of potential transverse TE materials. Furthermore, it paves the way for their integration into diverse TE engineering applications, mirroring the significant advancements in conventional TE research with structurally engineered materials.

## 4 Experimental Section

### Synthesis of SYCO Polycrystals

SYCO polycrystals were synthesized via a solid‐state reaction using SrCO_3_ (99.9%, Sigma Aldrich), Y_2_O_3_ (99.99%, Sigma Aldrich) and Co_3_O_4_ (99.5%, Sigma Aldrich) powders. First, the raw materials were mixed in stoichiometric ratios with 0, 3 and 5 mol% extra Co_3_O_4_ to introduce controlled levels of oxygen vacancies. The mixtures were then calcined at 1100 °C for 12 h in air to ensure proper phase formation. Next, the calcined products were uniaxially pressed into 12.7 mm diameter pellets at 155 MPa (Hydraulic press, Carver Inc.), followed by sintering at 1100 °C for 48 h in air.

### Characterization of Microstructure, Chemical Composition and Crystal Structure

The microstructures of SYCO samples were examined using secondary electron imaging with SEM (Apreo SEM, Thermo Scientific) at an accelerating voltage of 15 kV and a beam current of 5.5 nA. The chemical composition, particularly the Sr:Y:Co ratio, was determined via inductively coupled plasma optical emission spectrometry (ICP‐OES) (ARCOS FHM22, SPECTRO). Additionally, the crystal structures of the SYCO samples were examined by powder XRD over a 2*θ* range of 30° to 70° (D/Max‐2500, Rigaku). Here, phase constitutions and lattice constants were estimated using JADE software.

### Investigation of the Oxidation State of Co Ions

XANES spectroscopy was employed to assess the Co valence states in SYCO samples. Specifically, Co K‐edge XANES spectra were collected at UNIST‐PAL 6D beamline, Pohang Accelerator Laboratory, South Korea. A Si (111) double‐crystal monochromator, detuned by 15% to minimize high‐order harmonic contamination, was used for monochromatization. The beam energy was calibrated by placing Co foils downstream to precisely determine the edge position. For measurement, each sample was ground into fine powder, uniformly distributed, and sandwiched between two layers of Kapton tape. Reference materials, CoO (Co^2+^) and Co_3_O_4_ (Co^2.67+^) from Sigma‐Aldrich, were prepared using the same procedure. All measurements were performed in transmission mode at room temperature. XPS spectroscopy was also employed to analyze the oxidation states of Co ions in each sample (Nexsa G2, Thermo Fisher). Due to the surface‐sensitive nature of XPS, ground powders were used for characterization, assuming that the obtained XPS data represent the bulk characteristics of the SYCO polycrystals. Here, the electron binding energy scale was calibrated to the C‐1s peak at 284.8 eV.

### Magnetic Property Measurements

The temperature *T* and magnetic field *H* dependence of magnetic moment *M* were measured using a magnetic property measurement system (MPMS) (Quantum Design, Inc.). Field‐cooled magnetization under 1 T was monitored over a temperature range of 2–400 K, while isothermal magnetization was measured at 260 K across a field range of −1.2 to 1.2 T.

### Characterization of Atomic Structures

The atomic structures of *δ* = 0.02 and 0.14 samples were analyzed using STEM. STEM specimens were prepared using focused ion beam (FIB) milling and flat polishing. The FIB milling process was performed with a dual‐beam focused ion beam system (Helios G3, FEI) using a 30 kV Ga ion beam, followed by low‐energy cleaning at acceleration voltages ranging from 5 to 1 kV to minimize Ga ion‐induced damage. Flat polishing involved mechanical thinning to ≈10 µm, ensuring crack‐free conditions. Afterward, the specimens were ion‐milled with a 3 keV Ar^+^ ion beam (PIPS І, Gatan, USA) and further refined using a 0.1 keV Ar^+^ beam (PIPS II, Gatan, USA) to reduce surface damage. HAADF and ABF STEM imaging were conducted using a STEM system (JEM‐ARM200F, JEOL) operating at 200 kV, equipped with an aberration corrector (ASCOR, CEOS GmbH) at the Materials Imaging & Analysis Center, POSTECH, South Korea. The electron probe was optimized to ≈78 pm. The HAADF detector's collection semi‐angles ranged from 68 to 280 mrad, enabling Z‐sensitive imaging, while ABF images—optimized for light element detection—were acquired using a 3 mm aperture with a collection angle of 12 to 24 mrad. STEM images were processed using Smart Align (HREM Research Inc., Japan) for multi‐stacking and alignment through rigid registration, correcting for sample drift and scan distortions. A band‐pass Wiener filter (HREM Research Inc.) with a local window was applied to reduce background noise. Image analysis was performed in Python using customized atomic analysis code. Chemical classification of A, B, and O sites (A: Sr/Y and B: Co) was based on intensity variations and atomic row differences in the ABF‐STEM images. The unit cell was defined as a B‐site surrounded by four neighboring A‐sites.

### Electric and TE Property Measurements

The synthesized SYCO samples were cut into bars and slabs using a diamond wire saw (STX‐202A, MTI Korea) for TE transport property and anomalous Hall (or Nernst) measurements, respectively. Copper and constantan wire electrodes (0.001‐in. dia., SPCP/SPCI‐001‐50, Omega Engineering, Inc.) were attached to the side surfaces of each sample using silver epoxy (H20E, EPO‐TEK) or carbon paste. For bar samples, an insulating BeO pad was secured with silver epoxy as a heat sink due to its high thermal conductivity (≈370 W m^−1^ K^−1^),^[^ ^75^ ^]^ while a 120‐Ω resistive heater was attached to the top using the same adhesive. For slab samples, they were sandwiched between two insulting BeO pads with Apiezon N grease, and two 120 Ω resistive heaters were placed on the top pad. Each assembled sample block was mounted on the sample stage of a custom liquid‐nitrogen cryostat system (Lake Shore Cryotronics, Inc.). Raw signals were recorded using a nanovoltmeter (2182A, Keithley) and an AC resistance bridge (model 372, Lake Shore Cryotronics, Inc.). All measurements were performed over a temperature range of 80–300 K in 20 K increments under high vacuum conditions (< 10^−6^ torr).

### DFT Calculations

DFT calculations were performed using the Vienna Ab *initio* Simulation Package (VASP).^[^ ^76^, ^77^ ^]^ The Perdew‐Burke‐Ernzerhof (PBE) formulation of the generalized gradient approximation (GGA)^[^ ^78^ ^]^ was employed for the exchange‐correlation functional, along with projector augmented wave (PAW) pseudopotentials.^[^ ^79^ ^]^ The plane‐wave basis set cutoff energy was set to 520 eV, and a Monkhorst‐Pack 6× 6× 4 k‐point grid^[^ ^80^ ^]^ was used for Brillouin zone sampling. To account for strongly correlated *d*‐electrons of Co, a Hubbard *U* correction^[^ ^81^ ^]^ was applied with *U* = 5.237 eV and *J* = 0.807 eV.^[^ ^82^ ^]^ Structural optimizations were performed until the Hellmann‐Feynman forces on all atoms were reduced below 0.01 eV Å^−1^.

## Conflict of Interest

The authors declare no conflict of interest.

## Supporting information



Supporting Information

## Data Availability

The data that support the findings of this study are available in the supplementary material of this article.

## References

[1] B. Poudel , Q. Hao , Y. Ma , Y. Lan , A. Minnich , B. Yu , X. Yan , D. Wang , A. Muto , D. Vashaee , X. Chen , J. Liu , M. S. Dresselhaus , G. Chen , Z. Ren , Science 2008, 320, 634. 18356488 10.1126/science.1156446

[2] D. Wu , L.‐D. Zhao , S. Hao , Q. Jiang , F. Zheng , J. W. Doak , H. Wu , H. Chi , Y. Gelbstein , C. Uher , C. Wolverton , M. Kanatzidis , J. He , J. Am. Chem. Soc. 2014, 136, 11412. 25072797 10.1021/ja504896a

[3] W. Li , L. Zheng , B. Ge , S. Lin , X. Zhang , Z. Chen , Y. Chang , Y. Pei , Adv. Mater. 2017, 29, 1605887. 10.1002/adma.201605887 28247491

[4] H. Jin , J. P. Heremans , Phys. Rev. Mater. 2018, 2, 115401.

[5] B. Ge , H. Lee , J. Im , Y. Choi , S.‐Y. Kim , J. Y. Lee , S.‐P. Cho , Y.‐E. Sung , K.‐Y. Choi , C. Zhou , Z. Shi , I. Chung , Energy Environ. Sci. 2023, 16, 3994.

[6] Y. Pan , B. He , X. Feng , F. Li , D. Chen , U. Burkhardt , C. Felser , Nat. Mater. 2025, 24, 76. 39753855 10.1038/s41563-024-02059-9 PMC11698688

[7] S. R. Boona , H. Jin , S. Watzman , J. Appl. Phys. 2021, 130, 171101.

[8] K.‐i. Uchida , W. Zhou , Y. Sakuraba , Appl. Phys. Lett. 2021, 118, 140504.

[9] W. Zhou , K. Yamamoto , A. Miura , R. Iguchi , Y. Miura , K.‐i. Uchida , Y. Sakuraba , Nat. Mater. 2021, 20, 463. 33462463 10.1038/s41563-020-00884-2

[10] M. Y. Kim , S. J. Park , G.‐Y. Kim , S.‐Y. Choi , H. Jin , Energy Environ. Sci. 2021, 14, 3480.

[11] M. R. Scudder , B. He , Y. Wang , A. Rai , D. G. Cahill , W. Windl , J. P. Heremans , J. E. Goldberger , Energy Environ. Sci. 2021, 14, 4009.

[12] K.‐i. Uchida , J. P. Heremans , Joule 2022, 6, 2240.

[13] T. Feng , P. Wang , Z. Han , L. Zhou , Z. Wang , W. Zhang , Q. Liu , W. Liu , Energy Environ. Sci. 2023, 16, 1560.

[14] S. Wu , X. Wang , X. Mi , S. Zheng , K. Yang , Z. Zhou , H. Wang , G. Han , X. Lu , Y. Pan , G. Wang , X. Zhou , Adv. Energy Mater. 2024, 14, 2400184.

[15] T. Itoh , Y. Kozuka , T. Hirai , K.‐i. Uchida , Adv. Funct. Mater. 2024, 34, 2409557.

[16] A. A. S. Ibrahim , A. Bochmann , R. Löhnert , J. Töpfer , Adv. Funct. Mater. 2025, 35, 2413166.

[17] M. Mizuguchi , S. Nakatsuji , Sci. Technol. Adv. Mater. 2019, 20, 262. 30956732 10.1080/14686996.2019.1585143 PMC6442159

[18] M. Murata , K. Nagase , K. Aoyama , A. Yamamoto , Y. Sakuraba , iScience 2021, 24, 101967. 33458616 10.1016/j.isci.2020.101967 PMC7797944

[19] W. Nernst , Ann. Phys. 1887, 267, 760.

[20] H. Narita , M. Ikhlas , M. Kimata , A. A. Nugroho , S. Nakatsuji , Y. Otani , Appl. Phys. Lett. 2017, 111, 202404.

[21] Z. Yang , E. A. Codecido , J. Marquez , Y. Zheng , J. P. Heremans , R. C. Myers , AIP Adv. 2017, 7, 095017.

[22] W. Zhou , Y. Sakuraba , Appl. Phys. Express 2020, 13, 043001.

[23] H. Narita , T. Higo , M. Ikhlas , S. Nakatsuji , Y. Otani , Appl. Phys. Lett. 2020, 116, 072404.

[24] J. Wang , A. Miura , R. Modak , Y. K. Takahashi , K.‐i. Uchida , Sci. Rep. 2021, 11, 11228. 34045651 10.1038/s41598-021-90865-5 PMC8160345

[25] T.‐W. Weng , T.‐C. Chuang , D. Qu , S.‐Y. Huang , J. Magn. Magn. Mater. 2022, 563, 169892.

[26] K. M. Bang , S. J. Park , H. Yu , H. Jin , Appl. Energy 2024, 368, 123222.

[27] S. J. Park , K. M. Bang , J. Park , H. Jin , Appl. Therm. Eng. 2025, 265, 125555.

[28] N. W. Ashcroft , N. D. Mermin , Solid State Phys. 1976, 116, 217.

[29] S. N. Guin , P. Vir , Y. Zhang , N. Kumar , S. J. Watzman , C. Fu , E. Liu , K. Manna , W. Schnelle , J. Gooth , C. Shekhar , Y. Sun , C. Felser , Adv. Mater. 2019, 31, 1806622. 10.1002/adma.201806622 31044469

[30] L. Ding , J. Koo , L. Xu , X. Li , X. Lu , L. Zhao , Q. Wang , Q. Yin , H. Lei , B. Yan , Z. Zhu , K. Behnia , Phys. Rev. X 2019, 9, 041061.

[31] A. Sakai , Y. P. Mizuta , A. A. Nugroho , R. Sihombing , T. Koretsune , M.‐T. Suzuki , N. Takemori , R. Ishii , D. Nishio‐Hamane , R. Arita , P. Goswami , S. Nakatsuji , Nat. Phys. 2018, 14, 1119.

[32] S. N. Guin , K. Manna , J. Noky , S. J. Watzman , C. Fu , N. Kumar , W. Schnelle , C. Shekhar , Y. Sun , J. Gooth , C. Felser , NPG Asia Mater. 2019, 11, 16.

[33] A. Sakai , S. Minami , T. Koretsune , T. Chen , T. Higo , Y. Wang , T. Nomoto , M. Hirayama , S. Miwa , D. Nishio‐Hamane , F. Ishii , R. Arita , S. Nakatsuji , Nature 2020, 581, 53. 32376952 10.1038/s41586-020-2230-z

[34] T. Asaba , V. Ivanov , S. M. Thomas , S. Y. Savrasov , J. D. Thompson , E. D. Bauer , F. Ronning , Sci. Adv. 2021, 7, abf1467. 10.1126/sciadv.abf1467 PMC7997519 33771869

[35] M. Li , H. Pi , Y. Zhao , T. Lin , Q. Zhang , X. Hu , C. Xiong , Z. Qiu , L. Wang , Y. Zhang , J. Cai , W. Liu , J. Sun , F. Hu , L. Gu , H. Weng , Q. Wu , S. Wang , Y. Chen , B. Shen , Adv. Mater. 2023, 35, 2301339. 10.1002/adma.202301339 37308132

[36] M. Ikhlas , T. Tomita , T. Koretsune , M.‐T. Suzuki , D. Nishio‐Hamane , R. Arita , Y. Otani , S. Nakatsuji , Nat. Phys. 2017, 13, 1085.

[37] G.‐Y. Guo , T.‐C. Wang , Phys. Rev. B 2017, 96, 224415.

[38] Y. Pan , C. Le , B. He , S. J. Watzman , M. Yao , J. Gooth , J. P. Heremans , Y. Sun , C. Felser , Nat. Mater. 2022, 21, 203. 34811495 10.1038/s41563-021-01149-2 PMC8810386

[39] S. Yoshida , W. Kobayashi , T. Nakano , I. Terasaki , K. Matsubayashi , Y. Uwatoko , I. Grigoraviciute , M. Karppinen , H. Yamauchi , J. Phys. Soc. Jpn. 2009, 78, 094711.

[40] A. Modi , M. A Bhat , S. Bhattacharya , G. S. Okram , N. K. Gaur , J. Appl. Phys. 2018, 123, 205114.

[41] H. Takahashi , S. Ishiwata , R. Okazaki , Y. Yasui , I. Terasaki , Phys. Rev. B 2018, 98, 024405.

[42] S. Balamurugan , K. Yamaura , A. B. Karki , D. P. Young , M. Arai , E. Takayama‐Muromachi , Phys. Rev. B 2006, 74, 172406.

[43] S. Hu , W. Han , X. Li , M. Ye , Y. Lu , C. Jin , Q. Liu , J. Wang , J. He , C. Cazorla , Y. Zhu , L. Chen , Adv. Energy Mater. 2022, 12, 2201469.

[44] K. Ito , J. Wang , Y. Shimada , H. Sharma , M. Mizuguchi , K. Takanashi , J. Appl. Phys. 2022, 132, 133904.

[45] V. Bartůněk , Š. Huber , D. Sedmidubský , Z. Sofer , P. Šimek , O. Jankovský , Ceram. Int. 2014, 40, 12591.

[46] J. W. Edwards , H. L. Johnston , W. E. Ditmars , J. Am. Chem. Soc. 1951, 73, 4729.

[47] W. Kobayashi , S. Ishiwata , I. Terasaki , M. Takano , I. Grigoraviciute , H. Yamauchi , M. Karppinen , Phys. Rev. B 2005, 72, 104408.

[48] T. Kishida , M. D. Kapetanakis , J. Yan , B. C. Sales , S. T. Pantelides , S. J. Pennycook , M. F. Chisholm , Sci. Rep. 2016, 6, 19762. 26818899 10.1038/srep19762 PMC4730147

[49] O. Haas , R. P. W. J. Struis , J. M. McBreen , J. Solid State Chem. 2004, 177, 1000.

[50] M. S. Platunov , V. A. Dudnikov , Y. S. Orlov , N. V. Kazak , L. A. Solovyov , Y. V. Zubavichus , A. A. Veligzhanin , P. V. Dorovatovskii , S. N. Vereshchagin , K. A. Shaykhutdinov , S. G. Ovchinnikov , JETP Lett. 2016, 103, 196.

[51] S. Samira , J. Hong , J. C. A. Camayang , K. Sun , A. S. Hoffman , S. R. Bare , E. Nikolla , JACS Au 2021, 1, 2224. 34977894 10.1021/jacsau.1c00359 PMC8715492

[52] T. Tsuzuki , R. He , A. Dodd , M. Saunders , Nanomaterials 2019, 9, 481. 30934596 10.3390/nano9030481 PMC6474108

[53] S. Fukushima , T. Sato , D. Akahoshi , H. Kuwahara , J. Phys. Soc. Jpn. 2009, 78, 064706.

[54] M. C. Biesinger , B. P. Payne , A. P. Grosvenor , L. W. M. Lau , A. R. Gerson , R. S. C. Smart , Appl. Surf. Sci. 2011, 257, 2717.

[55] Y. Pan , X. Xu , Y. Zhong , L. Ge , Y. Chen , J.‐P. M. Veder , D. Guan , R. O'Hayre , M. Li , G. Wang , H. Wang , W. Zhou , Z. Shao , Nat. Commun. 2020, 11, 2002. 32332731 10.1038/s41467-020-15873-x PMC7181763

[56] Y.‐M. Kim , J. He , M. D. Biegalski , H. Ambaye , V. Lauter , H. M. Christen , S. T. Pantelides , S. J. Pennycook , S. V. Kalinin , A. Y. Borisevich , Nat. Mater. 2012, 11, 888. 22902896 10.1038/nmat3393

[57] S. Zhang , G. Galli , npj Comput. Mater. 2020, 6, 170.

[58] M. Soroka , K. Knížek , Z. Jirák , P. Levinský , M. Jarošová , J. Buršik , J. Hejtmánek , Phys. Rev. Mater. 2021, 5, 035401.

[59] M. Manikandan , A. Ghosh , R. Mahendiran , J. Phys. Chem. C 2022, 126, 1152.

[60] W.‐L. Lee , S. Watauchi , V. L. Miller , R. J. Cava , N. P. Ong , Phys. Rev. Lett. 2004, 93, 226601. 15601108 10.1103/PhysRevLett.93.226601

[61] W. Koshibae , K. Tsutsui , S. Maekawa , Phys. Rev. B 2000, 62, 6869.

[62] A. A. Taskin , A. N. Lavrov , Y. Ando , Phys. Rev. B 2006, 73, 121101.

[63] A. A. Burkov , L. Balents , Phys. Rev. Lett. 2003, 91, 057202. 12906629 10.1103/PhysRevLett.91.057202

[64] D. Xiao , Y. Yao , Z. Fang , Q. Niu , Phys. Rev. Lett. 2006, 97, 026603. 16907470 10.1103/PhysRevLett.97.026603

[65] Y. Pu , D. Chiba , F. Matsukura , H. Ohno , J. Shi , Phys. Rev. Lett. 2008, 101, 117208. 18851329 10.1103/PhysRevLett.101.117208

[66] S. Watanabe , M. Ohno , Y. Yamashita , T. Terashige , H. Okamoto , J. Takeya , Phys. Rev. B 2019, 100, 241201.

[67] V. Ambegaokar , B. I. Halperin , J. S. Langer , Phys. Rev. B 1971, 4, 2612.

[68] N. F. Mott , E. A. Davis , Electronic Processes in Noncrystalline Materials, Oxford University Press, Oxford 1979.

[69] A. Ali , A. Hassen , B. Kim , Y. Wu , B. G. Kim , S. Park , J. Korean Phys. Soc. 2007, 51, 1736.

[70] A. S. Ioselevich , Phys. Rev. Lett. 1995, 74, 1411. 10059013 10.1103/PhysRevLett.74.1411

[71] H. Wang , G. Li , X. Guan , M. Zhao , L. Li , Phys. Chem. Chem. Phys. 2011, 13, 17775. 21901200 10.1039/c1cp21562k

[72] J. Zhang , A. Visinoiu , F. Heyroth , F. Syrowatka , M. Alexe , D. Hesse , H. S. Leipner , Phys. Rev. B 2005, 71, 064108.

[73] D. Fuchs , E. Arac , C. Pinta , S. Schuppler , R. Schneider , H. v. Löhneysen , Phys. Rev. B 2008, 77, 014434.

[74] G. E. Sterbinsky , P. J. Ryan , J. W. Kim , E. Karapetrova , J. X. Ma , J. Shi , J. C. Woicik , Phys. Rev. B 2012, 85, 020403. 10.1103/PhysRevLett.120.197201 29799260

[75] G. A. Slack , S. B. Austerman , J. Appl. Phys. 1971, 42, 4713.

[76] G. Kresse , J. Furthmüller , Phys. Rev. B 1996, 54, 11169. 10.1103/physrevb.54.11169 9984901

[77] G. Kresse , J. Furthmüller , Comput. Mater. Sci. 1996, 6, 15.

[78] J. P. Perdew , K. Burke , M. Ernzerhof , Phys. Rev. Lett. 1996, 77, 3865. 10062328 10.1103/PhysRevLett.77.3865

[79] P. E. Blöchl , Phys. Rev. B 1994, 50, 17953. 10.1103/physrevb.50.17953 9976227

[80] H. J. Monkhorst , J. D. Pack , Phys. Rev. B 1976, 13, 5188.

[81] S. L. Dudarev , G. A. Botton , S. Y. Savrasov , C. J. Humphreys , A. P. Sutton , Phys. Rev. B 1998, 57, 1505.

[82] G. C. Moore , M. K. Horton , E. Linscott , A. M. Ganose , M. Siron , D. D. O'Regan , K. A. Persson , Phys. Rev. Mater. 2024, 8, 014409.

