# Supplementary material for: Oxygen Vacancy Defect Engineering for Transverse Thermoelectric Enhancement: a Novel Extrinsic Pathway beyond Intrinsic Approaches
Source: Adv Sci (Weinh). 2025 Apr 17;12(27):2502892. doi: 10.1002/advs.202502892 (PMC12279219; doi:10.1002/advs.202502892)
Supplement: Supplementary file 1 — Supporting Information [file ADVS-12-2502892-s001.docx]

Supporting Information

**Oxygen Vacancy Defect Engineering for Transverse Thermoelectric Enhancement: A Novel Extrinsic Pathway Beyond Intrinsic Approaches**

*Min Young Kim, Dongkyu Lee, June Ho Lee, Donghwa Lee, Gi-Yeop Kim, Si-Young Choi, Joseph P. Heremans and Hyungyu Jin**

1. **Crystal structure of Sr_3_YCo_4_O_10_**

**
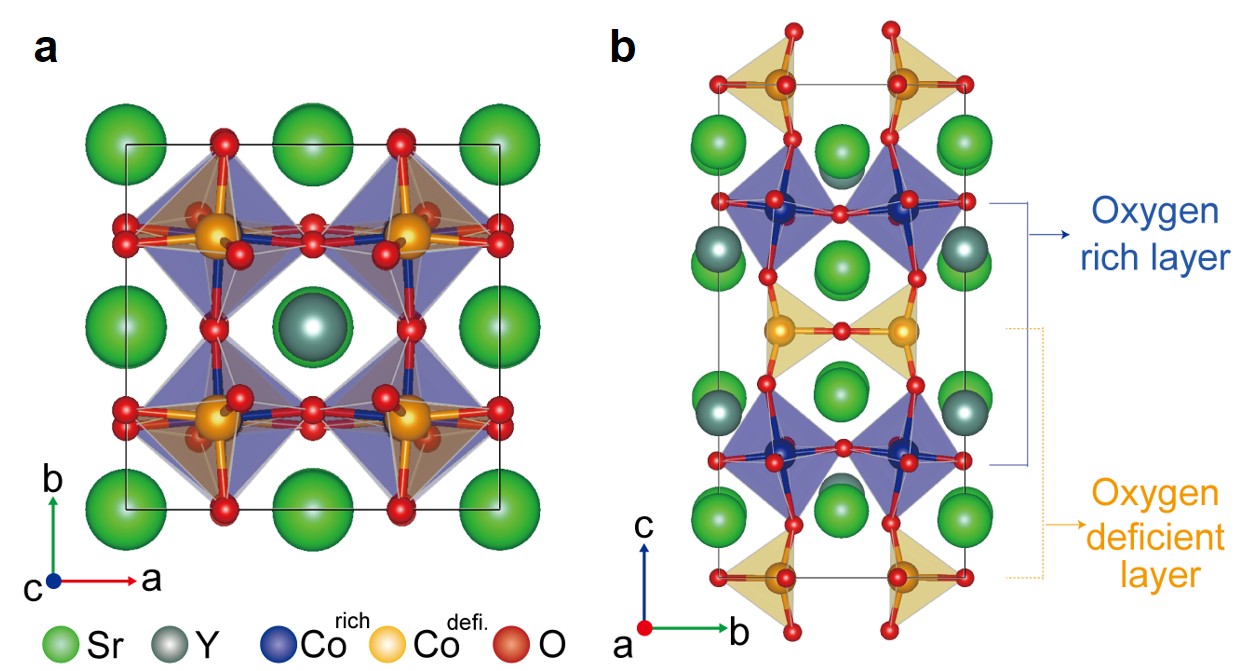
**

**Figure S1.** The unit cells of oxygen vacancy-ordered brownmillerite Sr_3_YCo_4_O_10_. (a) Top view: projection along the *c*-axis. (b) Side view: projection along the *a*-axis.

1. **Microstructural and chemical composition analysis**


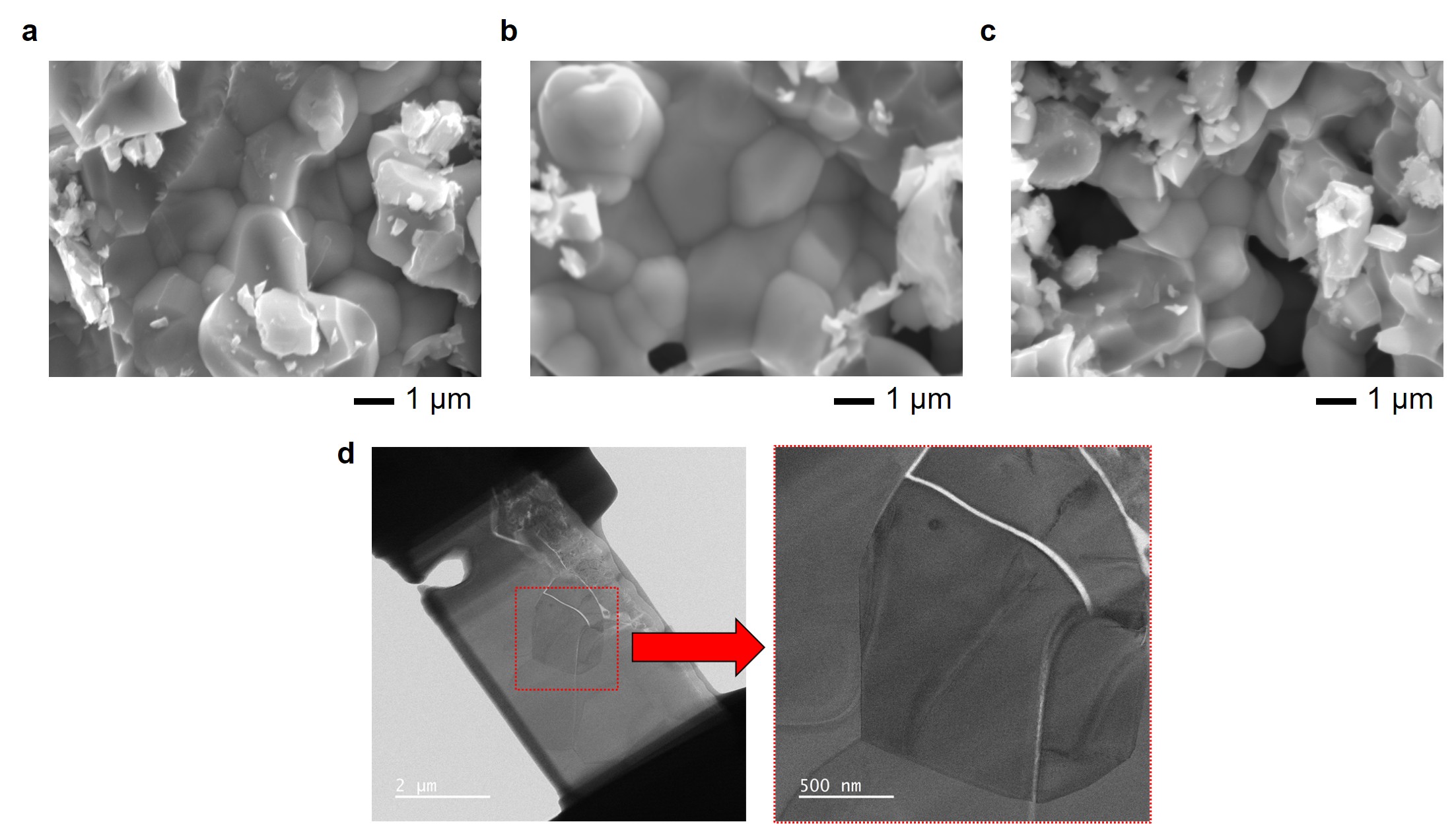


**Figure S2.** SEM images of SYCO polycrystals synthesized with (a) 0 mol%, (b) 3 mol% and (c) 5 mol% extra Co_3_O_4_. (d) Bright-field (BF) STEM image of the SYCO sample with 5 mol% added Co_3_O_4_.

**
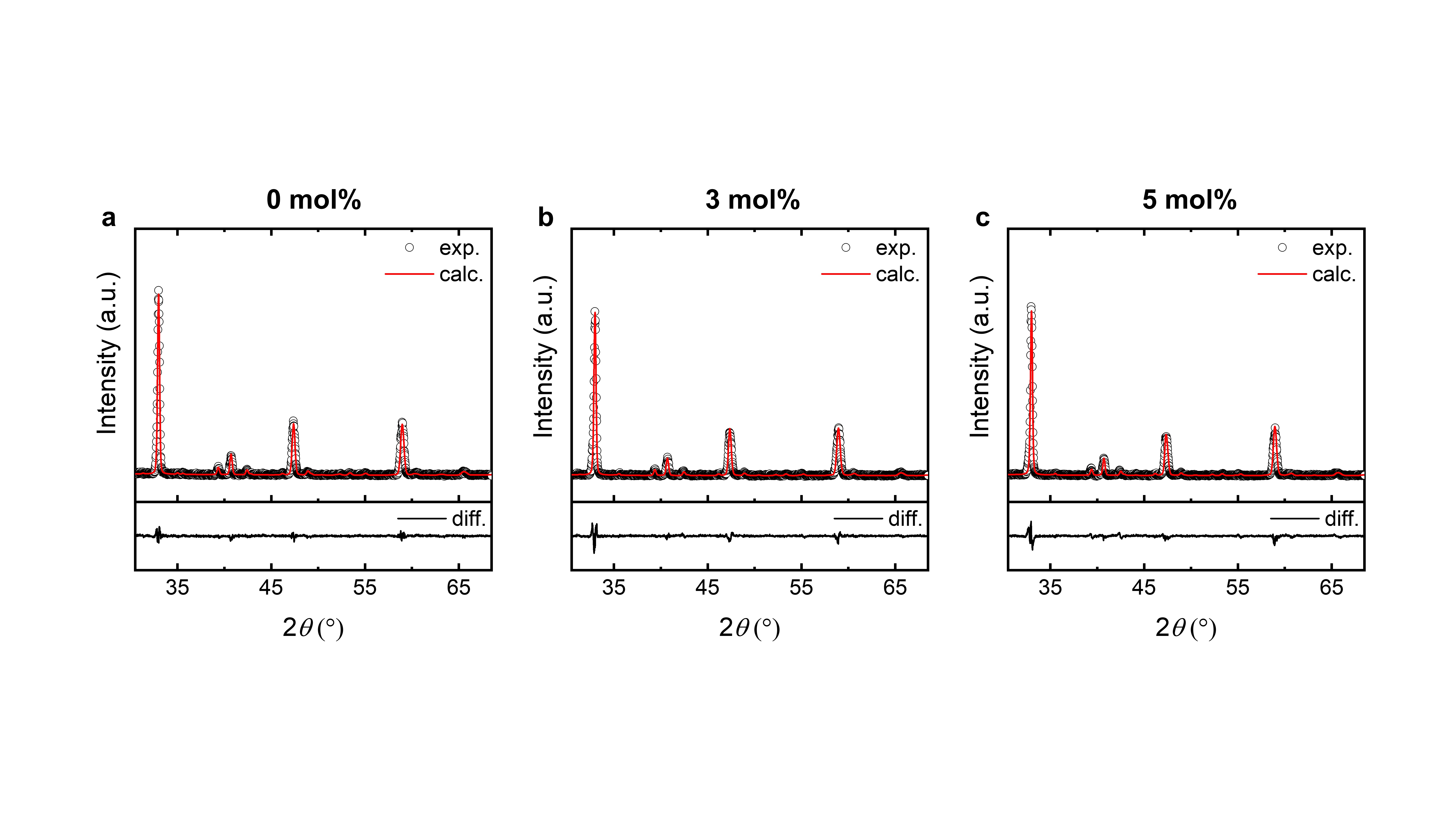
**

**Figure S3.** XRD refinement results of the SYCO samples synthesized with (a) 0, (b) 3, and (c) 5 mol% additional Co_3_O_4_.


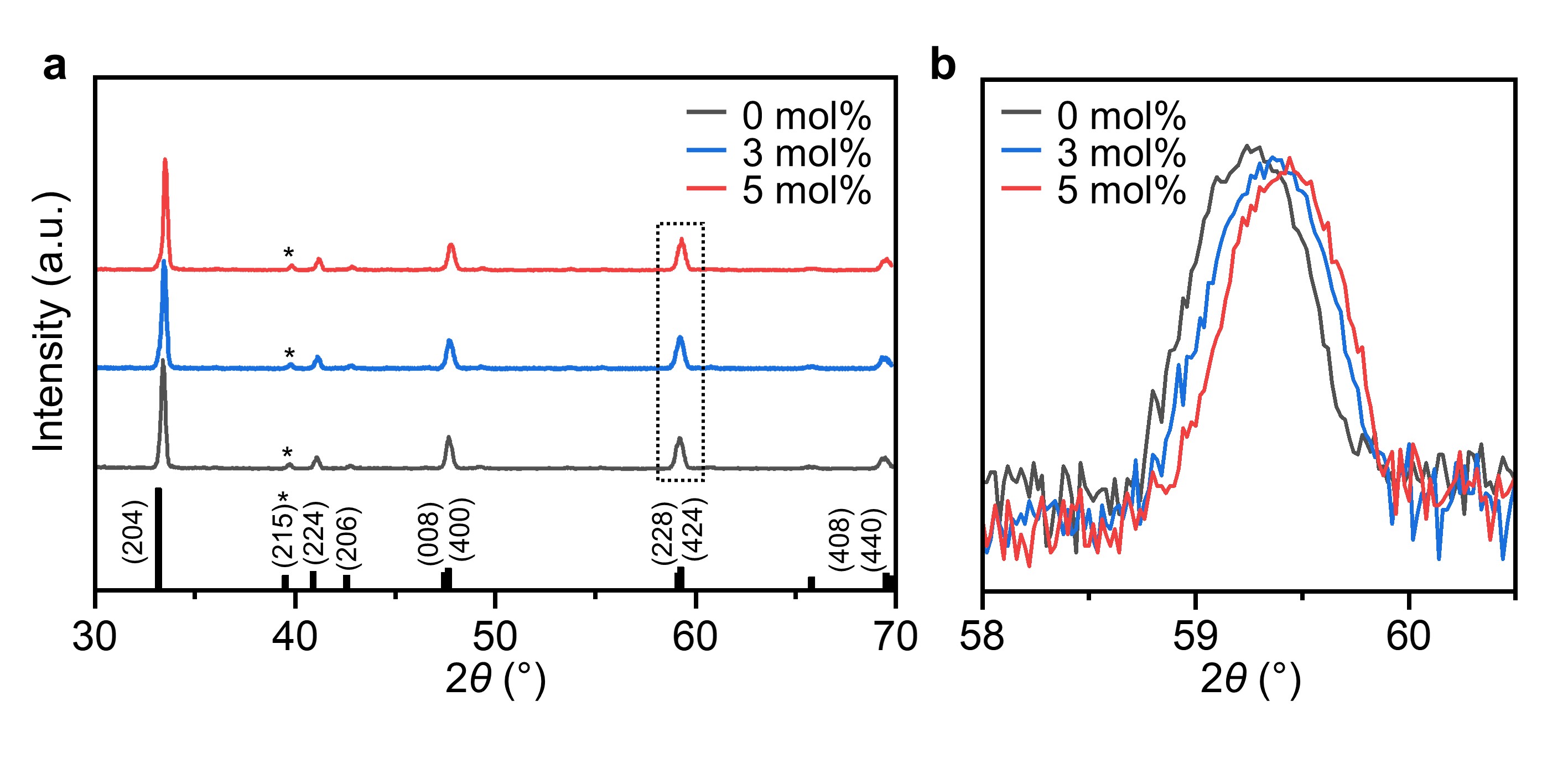


**Figure S4.** (a) XRD patterns of the SYCO samples synthesized with 0, 3, and 5 mol% additional Co_3_O_4_. (b) An enlarged view of (a) in the 2*θ* range from 58˚ to 60.5˚.


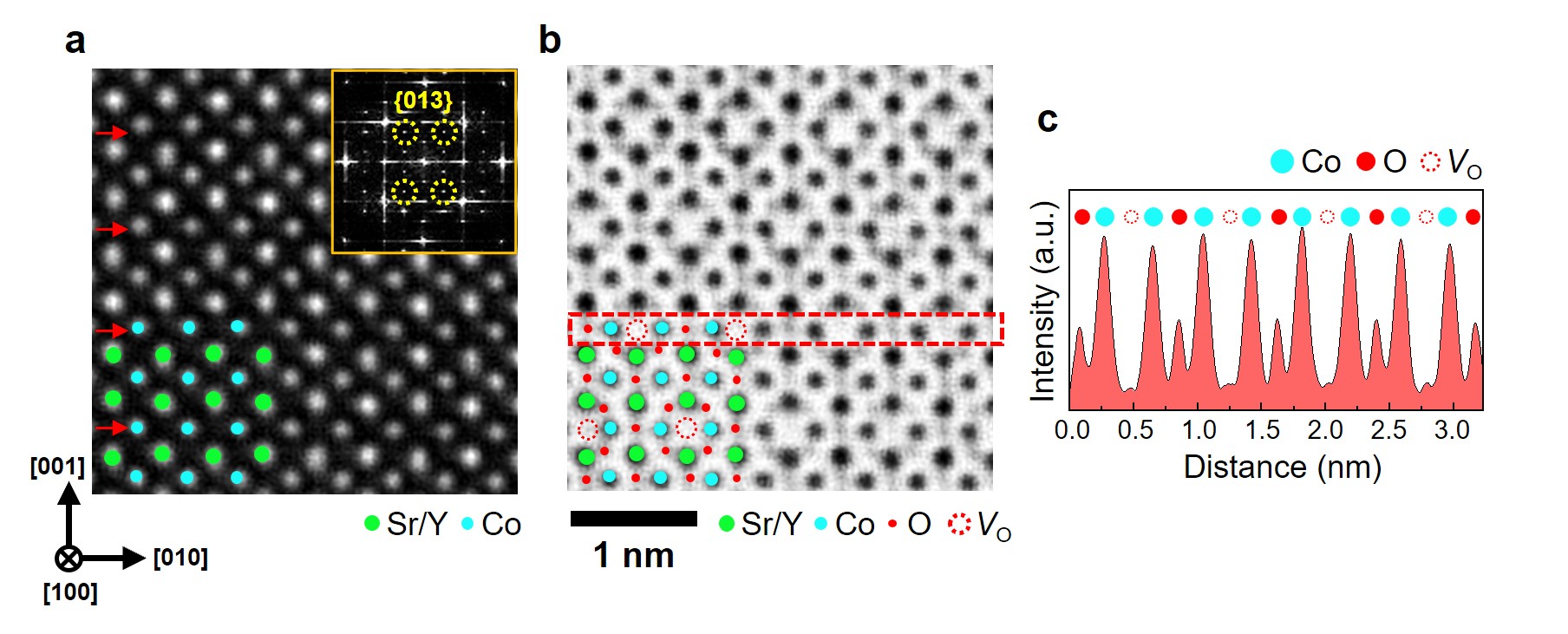


**Figure S5.** (a) HAADF-STEM and (b) ABF-STEM images of SYCO atomic structures synthesized with extra Co_3_O_4_ (5 mol%) along the [100] zone axis. Red arrows highlight the CoO_4.25+d_ layer. The inset in (a) shows the Fast Fourier Transform (FFT) patterns derived from the HAADF image, with yellow dotted circles indicating diffraction spots corresponding to the ordered superstructure of the {013} planes. (c) Intensity line profiles extracted from the red dotted box in the ABF-STEM image in (b).

**
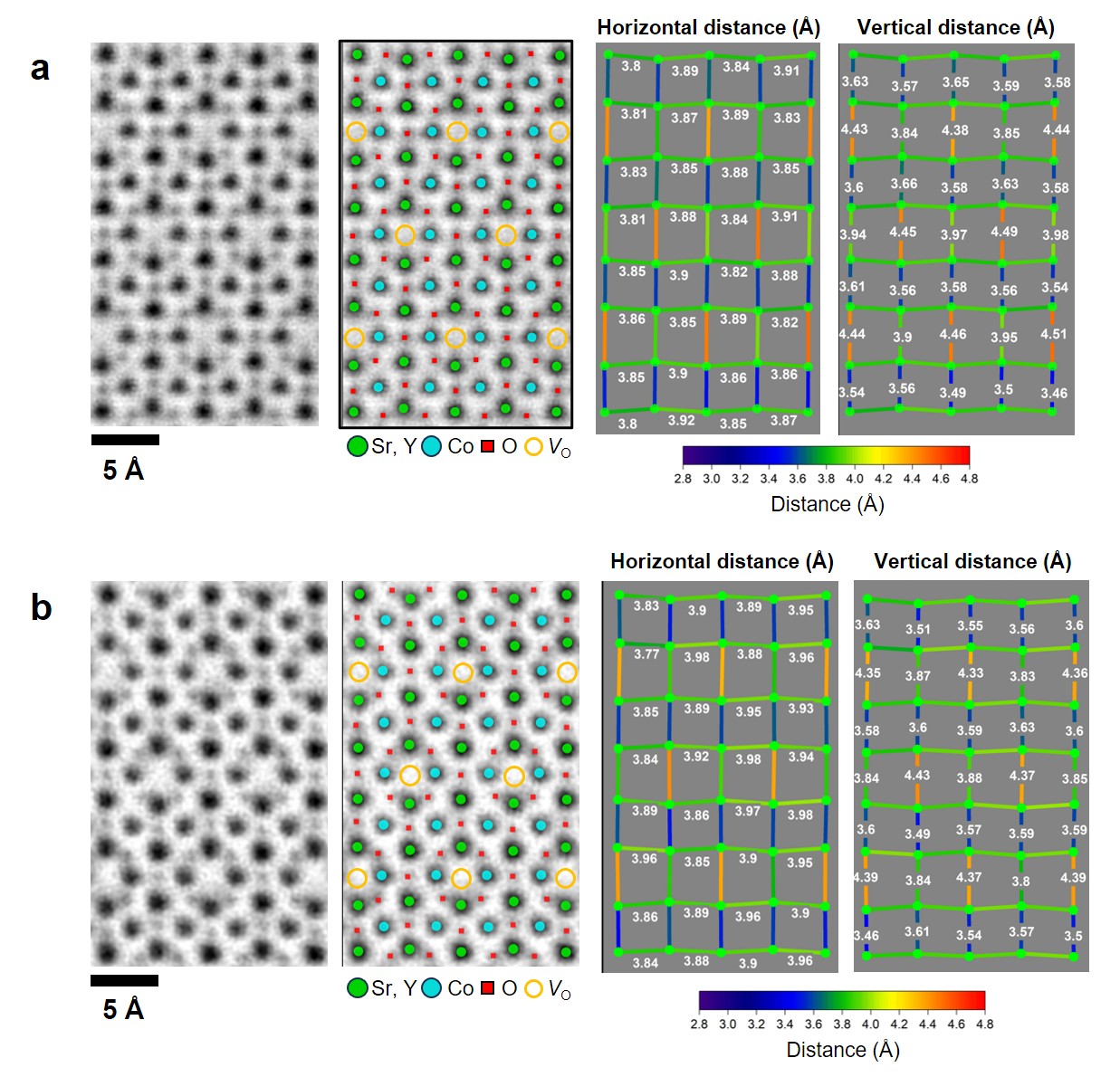
**

**Figure S6.** ABF-STEM image analysis of the A-site lattice distances in the SYCO polycrystal system along the horizontal and vertical directions. (a) ABF-STEM image lattice distance measures for the samples with (a) 0 mol% and (b) 5 mol% additional Co_3_O_4_.

**Table S1.** Chemical composition of SYCO polycrystals as a function of the added Co_3_O_4_ powder.

| Co_3_O_4_ (mol%) | Sr (at.%) | Y (at.%) | Co (at.%) | Sr:Y:Co |
| --- | --- | --- | --- | --- |
| 0 | 36.9 | 12.7 | 50.4 | 3 : 1.03 : 4.10 |
| 3 | 37.0 | 12.5 | 50.5 | 3 : 1.01 : 4.09 |
| 5 | 37.6 | 12.5 | 49.9 | 3 : 1.00 : 3.98 |

**Table S2.** *a*-axis and *c*-axis lattice constants *a* and *c* of the SYCO samples as a function of the added Co_3_O_4_ powder at room temperature.

| Co_3_O_4_ (mol%) | *a* (Å) | *c* (Å) | *c/a* | GoF |
| --- | --- | --- | --- | --- |
| 0 | 7.6659 | 15.367 | 2.005 | 1.56 |
| 3 | 7.6770 | 15.317 | 1.995 | 1.77 |
| 5 | 7.6812 | 15.304 | 1.992 | 1.96 |

The refinement is deemed academically reliable when the goodness-of-fit (GoF) value is below a certain threshold (GoF < 2).^[1]^

1. **Analysis of Co reduction using X-ray photoelectron spectroscopy (XPS)**

**
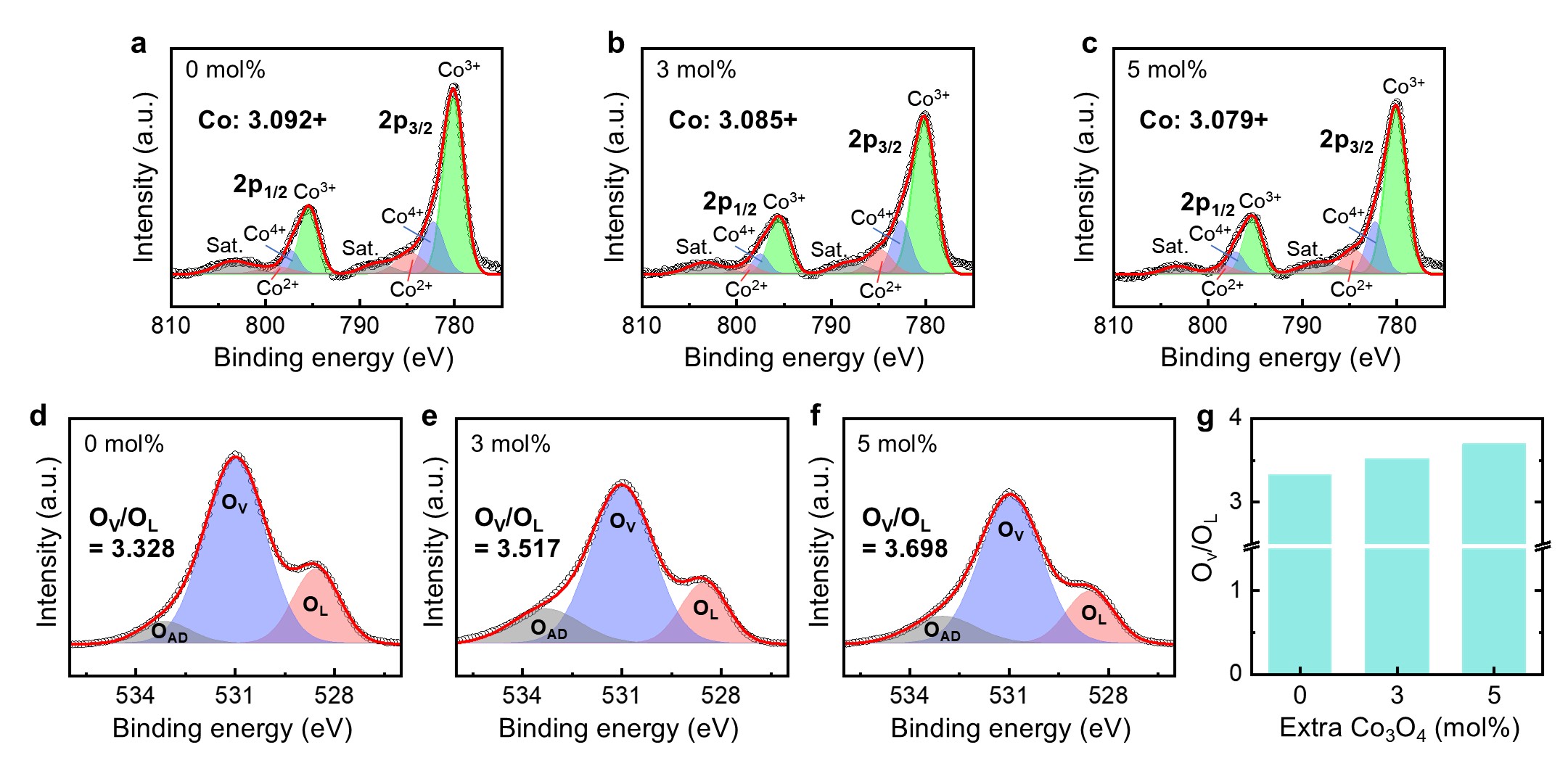
**

**Figure S7.** Analysis of Co reduction in the SYCO polycrystals using XPS. The Co-2p spectra of (a) 0, (b) 3, and (c) 5 mol% SYCO samples, along with peak fitting results based on mixed valence Co ions. The O-1s spectra of (d) 0, (e) 3, and (f) 5 mol% SYCO samples, showing deconvoluted peaks corresponding to chemisorbed O ions and Co-O bonds in Co_3_O_4_. (g) The ratio of the vacancy-related peak O_V_ to the lattice-related peak O_L_ as a function of the added Co_3_O_4_ content.

The oxidation states of Co ions in each sample were qualitatively analyzed using the Co-2p XPS spectra. As shown in **Fig. S7a-S7c**, all three spectra exhibit two prominent peaks at approximately 780 and 795 eV, corresponding to the spin-orbit split Co 2p_3/2_ and 2p_1/2_ states, respectively.^[2-4]^ To estimate the oxidation states of Co, the spectra were deconvoluted into peaks representing mixed-valence Co ions (Co^2+^, Co^3+^ and Co^4+^).^[3-4]^ Each peak position was carefully calibrated using the adventitious carbon peak at 284.8 eV, and Shirley background subtraction was applied for baseline correction.^[5]^ Additionally, satellite peaks, representing shake-up processes, were fitted at ~789.5 eV (2p_3/2_) and ~804.5 eV (2p_1/2_) to improve the fit quality, achieving *R*^2^ > 0.99 for all samples.^[2]^ The XPS curve-fitting analysis reveals that Co^3+^ ions account for approximately 70% of the total Co ions, with Co^4+^ and Co^2+^ contributing around 20% and 10%, respectively. Notably, the proportion of Co^3+^ increases as Co^4+^ ions decrease with increasing *δ*. This redistribution allows a qualitative estimation of the average oxidation states of Co ions in each sample: Co^3.092+^, Co^3.085+^ and Co^3.079+^ for the 0, 3 and 5 mol% SYCO samples, respectively. Similarly, the O-1s spectra were deconvoluted into two peaks near 528 and 531 eV, corresponding to chemisorbed oxygen ions and Co-O bonds in Co_3_O_4_, respectively (**Fig. S7d-S7f**).^[6]^ Furthermore, the increasing ratio of the vacancy-related peak *O*_V_ to the lattice-related peak *O*_L_ (**Fig. S7g**) corroborates the rising oxygen vacancy concentration, further supporting the observed decrease in the oxidation state of Co ions.

1. **Electrical and thermoelectric transport property measurements**


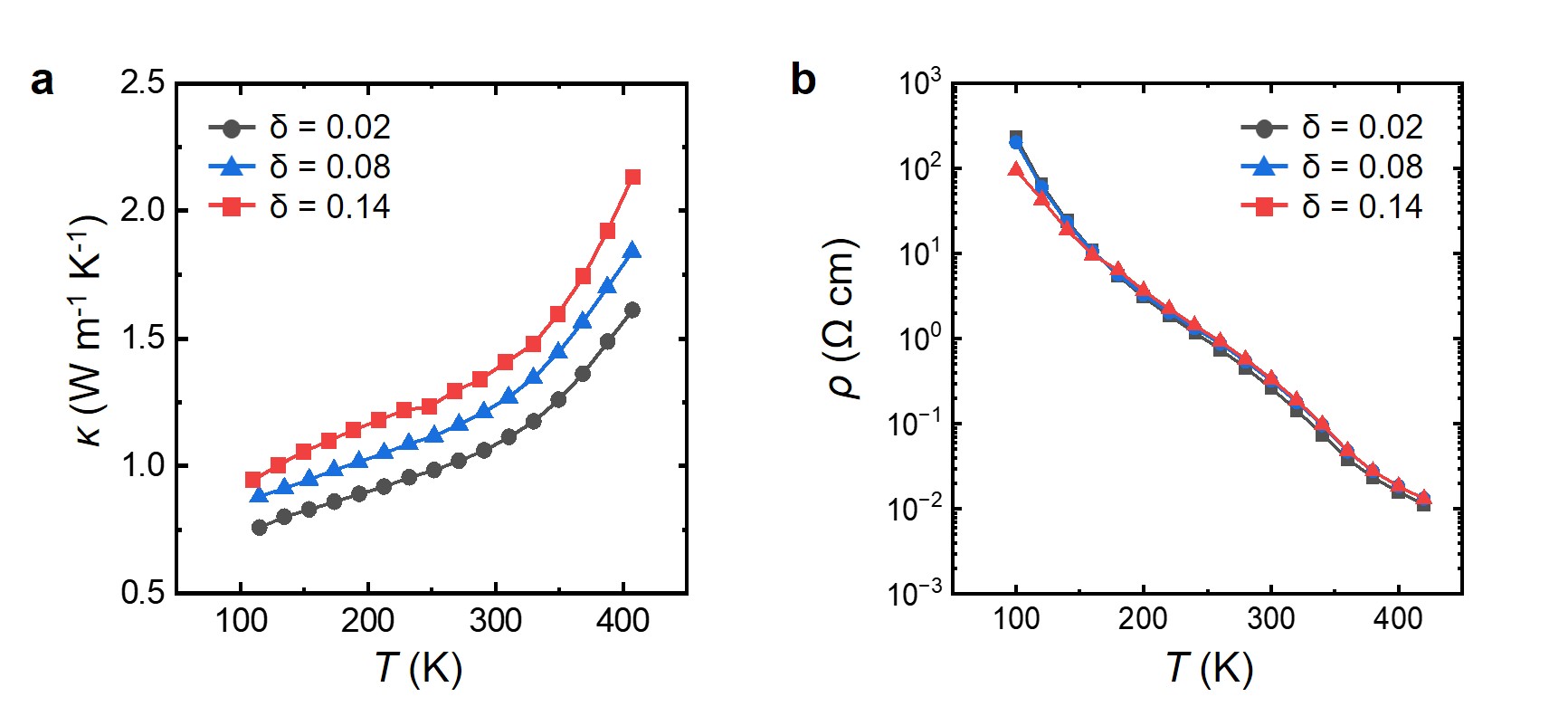


**Figure S8.** Temperature (*T*) dependence of (a) thermal conductivity *κ* and (b) electrical resistivity *ρ* for all SYCO samples.

Across all samples, the resistivity *ρ* increases by four orders of magnitude as *T* decreases. In particular, *ρ* below 160 K decrease with increasing *δ*, despite the reduction in total hole density resulting from the lower concentration of Co^4+^ ions.


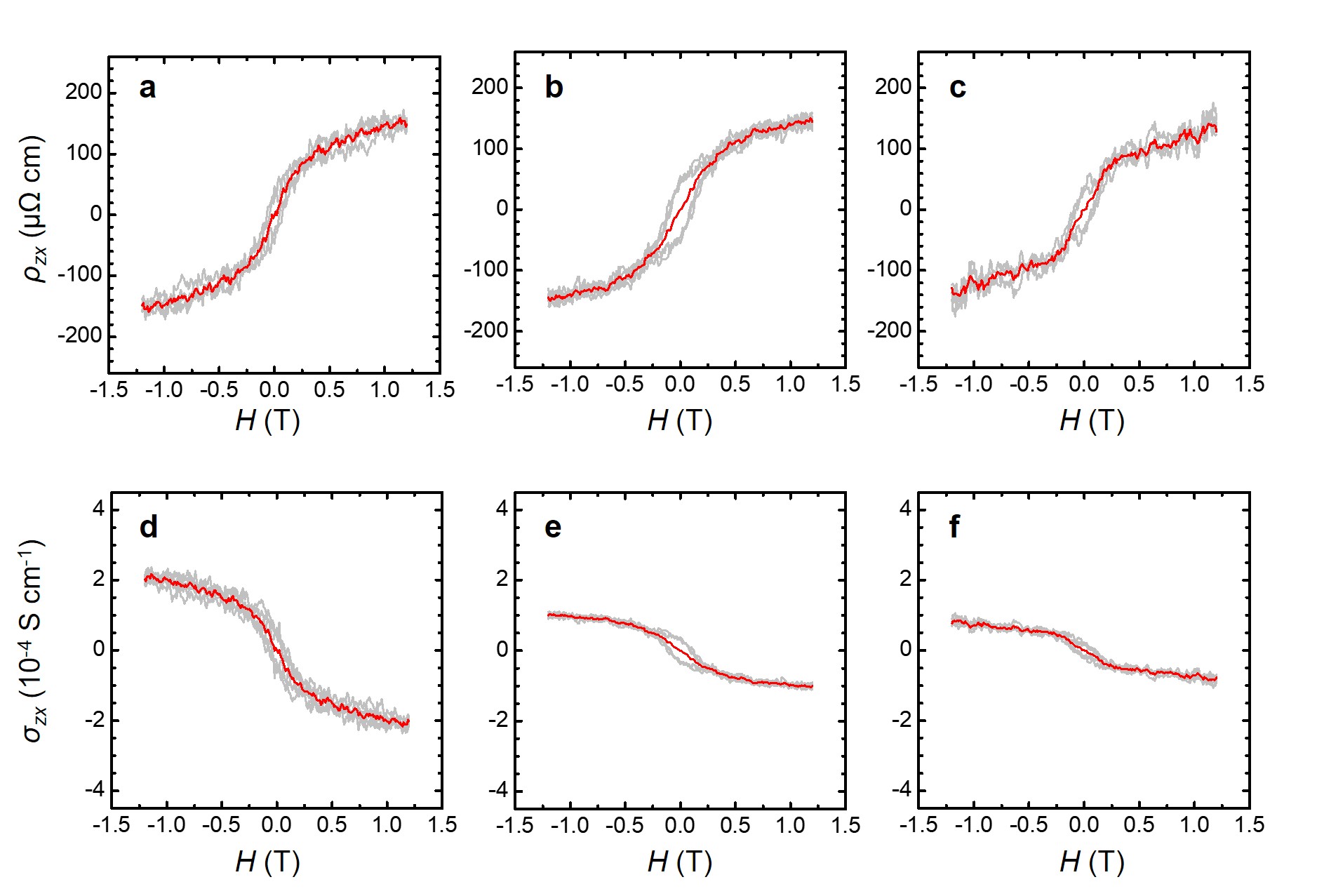


**Figure S9.** Magnetic field (*H*) dependence of (a-c) transverse electrical resistivity (*ρ_zx_*) and (d-f) transverse electrical conductivity (*σ_zx_*) at 260 K for samples with (a,d) *δ* = 0.02, (b,e) *δ* = 0.08 and, (c,f) *δ* = 0.14. The red lines represent the averaged values over three datasets.

*θ*_AHE_ and *θ*_ANE_ were calculated using the measured transport properties at 260 K based on the relationships $\theta_{\text{AHE}}\approx-\rho_{zx}/\rho_{xx}$ and $\theta_{\text{ANE}}\approx\frac{\alpha_{zx}}{\alpha_{xx}}=\frac{S_{\text{ANE}}}{S_{\text{Seebeck}}}-\frac{\rho_{zx}}{\rho_{xx}}$. Specifically, *ρ_zx_* at 1.2T was used for the calculations. The signals appear somewhat noisy due to the poor electrical conductivity of the SYCO samples and the presence of a Schottky barrier between the SYCO samples and electrical contacts. To achieve sufficient resolution, we averaged three datasets.

1. **Spin-state distribution of Co ions in the SYCO system**

**
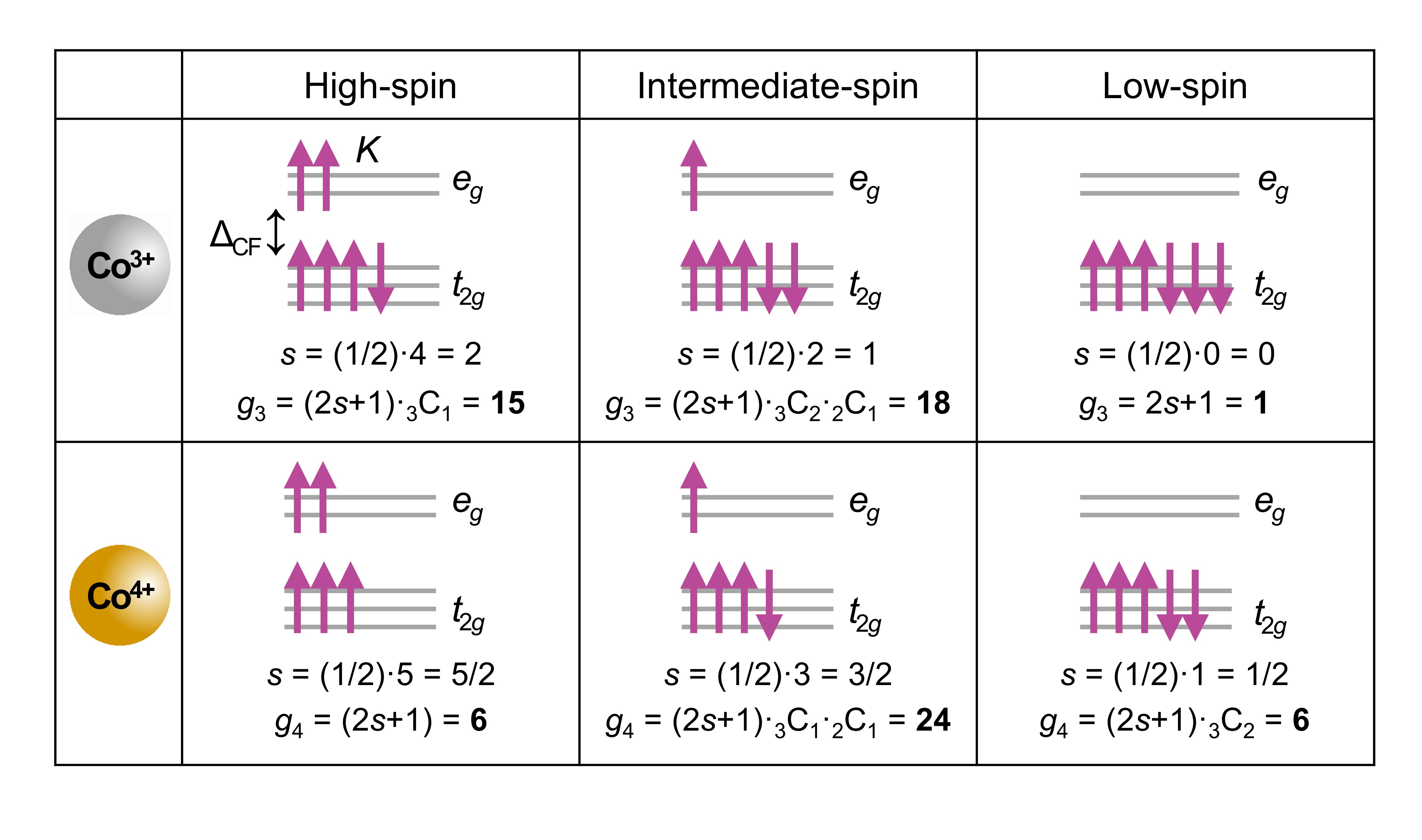
**

**Figure S10.** Schematic representation of the spin-state distribution of Co^3+^ and Co^4+^ ions, governed by Hund exchange energy (*K*) and crystal-field splitting (Δ_CF_). The lines represent the energy levels of the *e_g_* and *t*_2_*_g_* orbitals. *s* denotes the spin magnitude, and *g*_3_(*g*_4_) represents the spin-orbital degeneracy of Co^3+^(Co^4+^) ions.

Co ions tend to adopt lower spin states as the crystal-field splitting Δ_CF_ increases. Based on the magnitude of Δ_CF_, the spin states are classified into three distinct categories: low-spin (LS), intermediate-spin (IS), and high-spin (HS) states.

1. Electronic band structures of Sr_3_YCo_4_O_10_ and Sr_3_YCo_4_O_11_

**
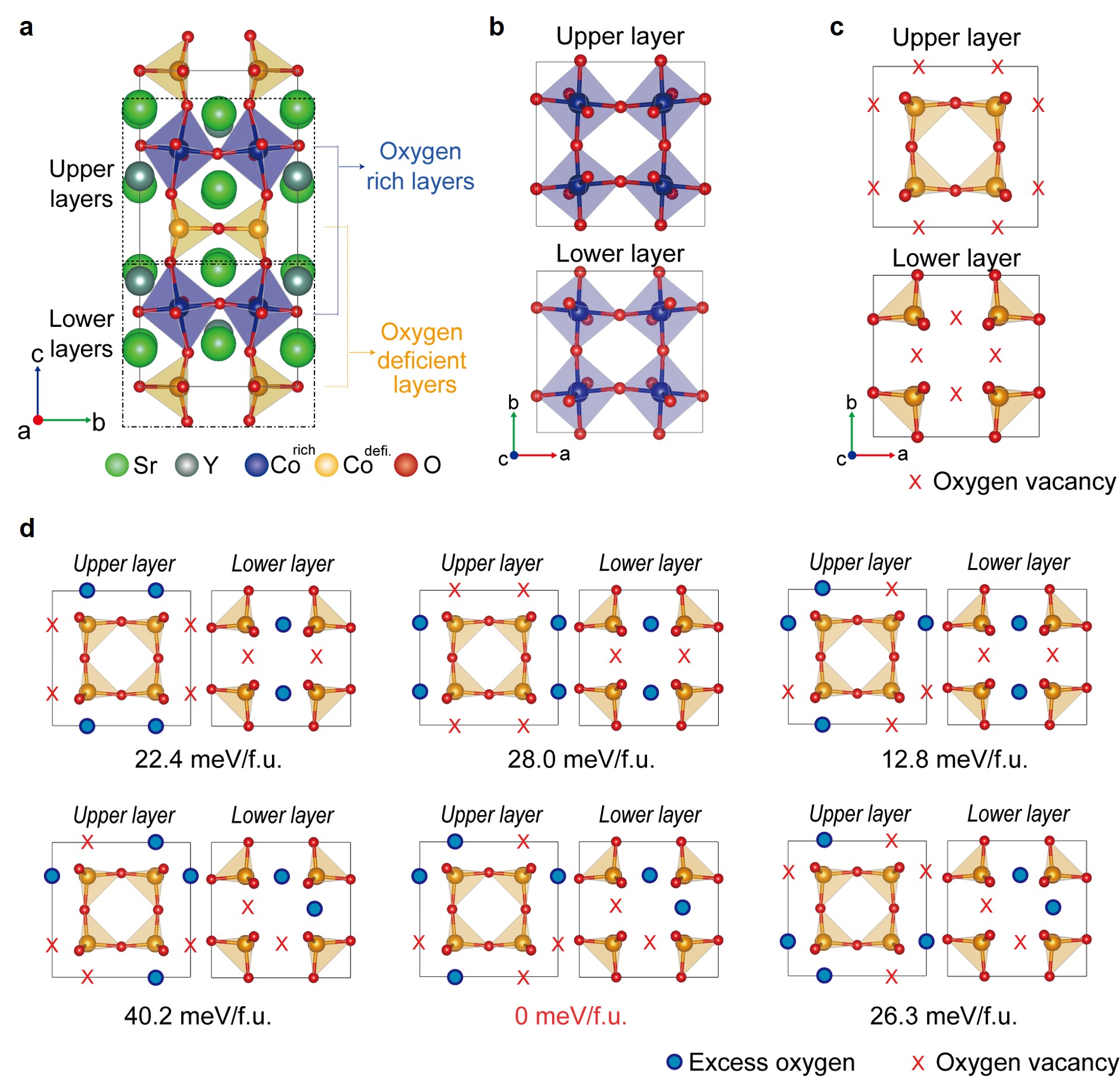
**

**Figure S11.** Structural comparison of oxygen vacancy-ordered brownmillerite Sr_3_YCo_4_O_10_ and oxygen-enriched Sr_3_YCo_4_O_11_. (a) Side view of Sr_3_YCo_4_O_10_, illustrating its layered framework. (b), (c) Top views of the upper and lower layers in Sr_3_YCo_4_O_10_: (b) the O-rich CoO_6_ layers and (c) the O-deficient CoO_4.25+d_ layers, where red ‘×’ symbols indicate the position of oxygen vacancies. (d) Six possible configurations of the four additional oxygen atoms necessary to form Sr_3_YCo_4_O_11_, along with their relative formation energies.

Since the Sr_3_YCo_4_O_11_ structure consists of four formula units along *c*-axis in unit cell, it incorporates four additional oxygen atoms compared to the perfect brownmillerite Sr_3_YCo_4_O_10_. These extra oxygen atoms are likely introduced into the O-deficient CoO_4.25+d_ layers, modifying the local coordination environment. To determine the most probable arrangement, six distinct arrangements were considered, and their relative formation energies were calculated. Among these, the most energetically favorable structure was identified and selected for further electronic structure analysis.

**
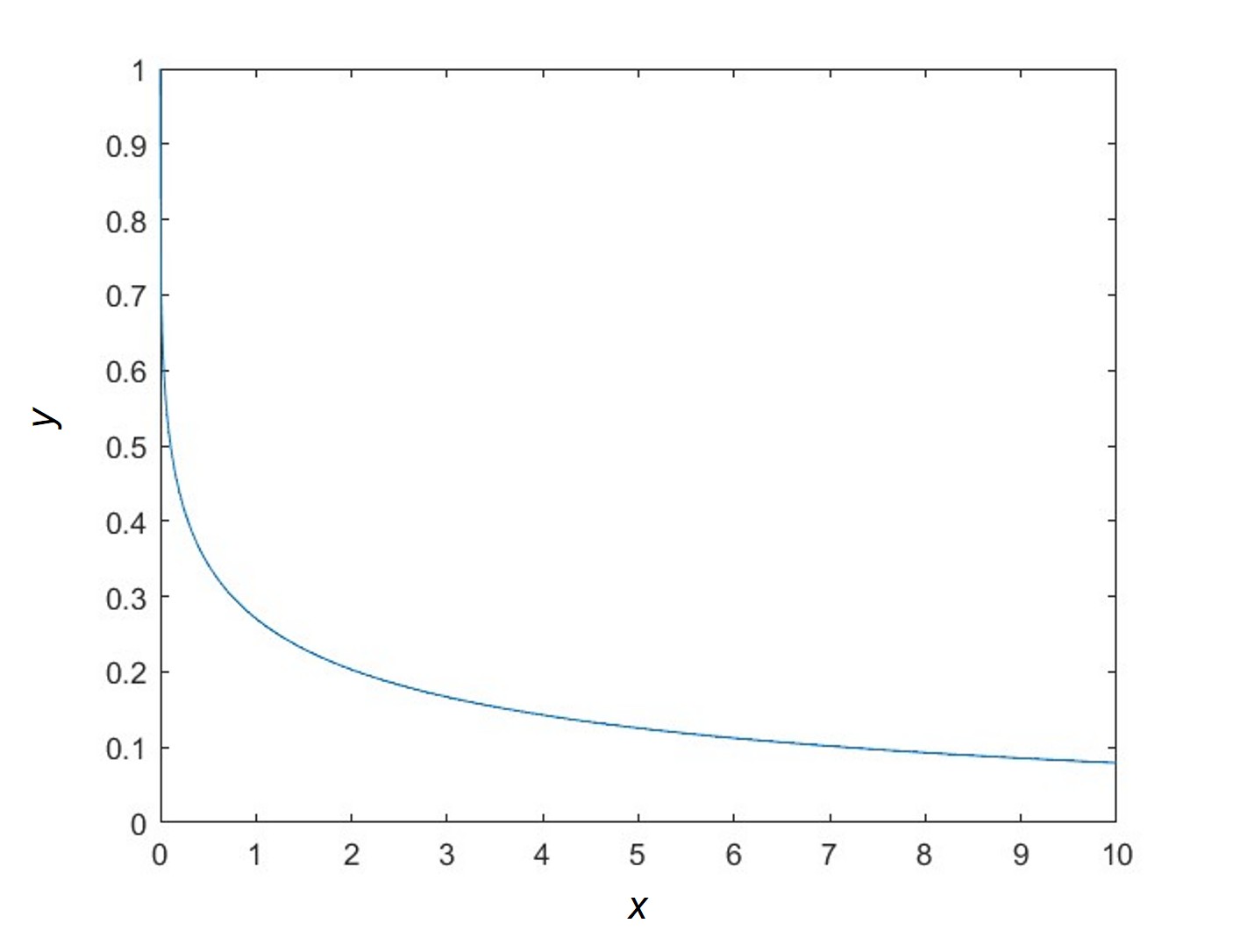
**

**Figure S12.** Graph for $y=e^{-2x^{1/4}}\left(x^{1/4}+1\right)$

**References**

[1] S. Y. Purwaningsih, N. Rosidah, M. Zainuri, T. Triwikantoro, S. Pratapa, D. Darminto, *J. Phys. Conf. Ser.* **2019**, 1153, 012070.

[2] T. J. Chuang, C. R. Brundle, D. W. Rice, *Surface Science* **1976**, 59, 413.

[3] M. C. Biesinger, B. P. Payne, A. P. Grosvenor, L. W. M. Lau, A. R. Gerson, R. S. C. Smart, *Applied Surface Science* **2011**, 257, 2717.

[4] Y. Pan, X. Xu, Y. Zhong, L. Ge, Y. Chen, J.-P. M. Veder, D. Guan, R. O’Hayre, M. Li, G. Wang, H. Wang, W. Zhou, Z. Shao, *Nature Communications* **2020**, 11, 2002.

[5] M. H. Engelhard, D. R. Baer, A. Herrera-Gomez, P. M. A. Sherwood, *Journal of Vacuum Science & Technology A* **2020**, 38.

[6] P. Srinivasan, A. J. Kulandaisamy, G. K. Mani, K. J. Babu, K. Tsuchiya, J. B. B. Rayappan, *RSC Advances* **2019**, 9, 30226.
